# Bruton’s Tyrosine Kinase-Mediated Signaling in Myeloid Cells Is Required for Protective Innate Immunity During Pneumococcal Pneumonia

**DOI:** 10.3389/fimmu.2021.723967

**Published:** 2021-09-06

**Authors:** Alexander P. de Porto, Zhe Liu, Regina de Beer, Sandrine Florquin, Joris J. T. H. Roelofs, Onno J. de Boer, Joke M. M. den Haan, Rudi W. Hendriks, Cornelis van ‘t Veer, Tom van der Poll, Alex F. de Vos

**Affiliations:** ^1^Center for Experimental and Molecular Medicine (CEMM), Amsterdam University Medical Centers (UMC), Academic Medical Center, University of Amsterdam, Amsterdam, Netherlands; ^2^Amsterdam Infection and Immunity Institute (AI&II), Amsterdam University Medical Centers (UMC), Amsterdam, Netherlands; ^3^Department of Pathology, Amsterdam University Medical Centers (UMC), Academic Medical Center, University of Amsterdam, Amsterdam, Netherlands; ^4^Department of Molecular Cell Biology and Immunology, Amsterdam University Medical Centers (UMC), Vrije Universiteit Amsterdam, Amsterdam, Netherlands; ^5^Department of Pulmonary Medicine, Erasmus Medical Center Rotterdam, University Medical Center, Rotterdam, Netherlands; ^6^Division of Infectious Diseases, Amsterdam University Medical Centers (UMC), Academic Medical Center, University of Amsterdam, Amsterdam, Netherlands

**Keywords:** BTK - Bruton’s tyrosine kinase, X-linked immunodeficiency, natural antibodies, Streptococcus pneumoniae, pneumonia, sepsis, myeloid cells, mice

## Abstract

Bruton’s tyrosine kinase (Btk) is a cytoplasmic kinase expressed in B cells and myeloid cells. It is essential for B cell development and natural antibody-mediated host defense against bacteria in humans and mice, but little is known about the role of Btk in innate host defense *in vivo*. Previous studies have indicated that lack of (natural) antibodies is paramount for impaired host defense against *Streptococcus (S.) pneumoniae* in patients and mice with a deficiency in functional Btk. In the present study, we re-examined the role of Btk in B cells and myeloid cells during pneumococcal pneumonia and sepsis in mice. The antibacterial defense of Btk^-/-^ mice was severely impaired during pneumococcal pneumosepsis and restoration of natural antibody production in Btk^-/-^ mice by transgenic expression of Btk specifically in B cells did not suffice to protect against infection. Btk^-/-^ mice with reinforced Btk expression in MhcII^+^ cells, including B cells, dendritic cells and macrophages, showed improved antibacterial defense as compared to Btk^-/-^ mice. Bacterial outgrowth in Lysmcre-Btk^fl^/Y mice was unaltered despite a reduced capacity of Btk-deficient alveolar macrophages to respond to pneumococci. Mrp8cre-Btk^fl^/Y mice with a neutrophil specific paucity in Btk expression, however, demonstrated impaired antibacterial defense. Neutrophils of Mrp8cre-Btk^fl^/Y mice displayed reduced release of granule content after pulmonary installation of lipoteichoic acid, a gram-positive bacterial cell wall component relevant for pneumococci. Moreover, Btk deficient neutrophils showed impaired degranulation and phagocytosis upon incubation with pneumococci *ex vivo*. Taken together, the results of our study indicate that besides regulating B cell-mediated immunity, Btk is critical for regulation of myeloid cell-mediated, and particularly neutrophil-mediated, innate host defense against *S. pneumoniae in vivo*.

## Introduction

Bruton’s tyrosine kinase (Btk) is a versatile signaling protein belonging to the Tyrosine kinase Expressed in hepatocellular Carcinoma (TEC) family and is expressed in the hematopoietic lineage, except in T cells and plasma cells (1–3). Btk is commonly known for its essential role in B cell development and function (4), and has gained attention as a very effective target to treat B cell malignancies (5). In humans, mutations in the Btk gene leading to absent or dysfunctional Btk expression cause the primary immunodeficiency X-linked agammaglobulinemia (XLA), characterized by an almost complete absence of peripheral B cells and immunoglobulins. Defective Btk signaling in mice results in a milder phenotype with abrogated follicular type 1 (IgM^low^IgD^hi^) and B-1 B cells development resulting in absent natural IgM and IgG_3_ antibodies (6, 7). Humans and mice with defective Btk signaling and patients taking Btk inhibitors are highly susceptibility to bacterial infections, especially with *Streptococcus (S.) pneumoniae* as causative microorganism (8–12). Treatment with intravenous immunoglobulins (IVIG) has significantly improved the prognosis of patients with XLA (13) which has led to the dogma that the role of Btk in myeloid cells is insignificant. This dogma was supported by the finding that X-linked immune deficiency (Xid) mice, which carry a mutation in the Btk gene leading to defective Btk signaling, were able to completely clear intravenously administered *S. pneumoniae* preincubated with natural antibodies against pneumococcal cell wall components such as phosphocholine (10, 14).

Multiple Btk-dependent responses, however, have also been described in macrophages, dendritic cells and neutrophils (15, 16). For example, Btk amplifies signaling of various Toll-like receptors (TLR), NLR family pyrin domain containing 3 (NLRP3), Dectin-1, Fc receptors and triggering receptor expressed on myeloid cells 1 (TREM-1) (17–20) and abrogated Btk signaling results in altered cytokine secretion, macrophage polarization and phagocytosis (16). Moreover, Btk has been described to aid in neutrophil development, migration and expression of granular proteins (21–23). These findings suggest that Btk signaling in myeloid cells is involved in host defense against bacterial infections. Additionally, a considerable fraction of XLA patients experience recurrent respiratory tract infections requiring hospital admission despite IVIG treatment (9). Moreover, patients treated with the irreversible Btk inhibitor Ibrutinib have normal levels of circulating immunoglobulins but are nevertheless prone to bacterial infection (24). To better understand why the absence of Btk signaling leads to an increased susceptibility for bacterial respiratory tract infections it is essential to improve our understanding of myeloid Btk function in antibacterial defense against invading pathogens.

We here investigated the role of Btk in the innate host defense against *S. pneumoniae* using well established models of pneumonia and sepsis (14, 25) in Btk deficient (Btk^-/-^) mice, Btk^-/-^ mice in which Btk expression is exclusively restored in B cells or in MhcII^+^ cells by a Btk transgene and in mice with a targeted deletion of the Btk gene specifically in myeloid cells (**Table S1**). The results of our study reveal that natural antibodies provide (only) partial protection to pneumosepsis evoked by *S. pneumoniae* and that Btk in myeloid cells, particularly in neutrophils, is also required for host defense against this pathogen.

## Materials and Methods

### Animals

All animals were housed in the Animal Research Institute Amsterdam under standard care. All mice were on a C57Bl/6 background. Specific pathogen-free wild type (WT) mice were bought from Charles River. Btk^-/-^, Cd19-Btk^+^ and MhcII-Btk^+^ mice, harboring a human Btk transgene, were previously described (6, 26, 27) and the latter two strains were maintained on a Btk^-/-^ background. Btk^fl/fl^ mice were generated from Btk^tm1a^ embryos (EUCOMM) as described previously (28). Female Btk^fl/fl^ mice were bred with male heterozygous Lysmcre mice (B6.129P2-Lyz2tm1(cre)Ifo/J; Jax stock number 004781) (29), Mrp8cre mice (B6.Cg-Tg(S100A8-cre,-EGFP)1Ilw/J; Jax stock number 021614) (30) and Cd19cre (B6N.129P2-Cd19tm1(cre)Cgn/J; Jax stock number 018958) transgenic mice (expressing Cre recombinase under the Lysm, Mrp8 and Cd19 promoter respectively; Jackson Laboratory) to generate male Lysmcre-Btk^fl^/Y, Mrp8cre-Btk^fl^/Y and Cd19cre-Btk^fl^/Y mice with specific Btk deletion and male Btk^fl^/Y as littermate controls. Only male mice were used for experiments. All experiments were carried out in accordance with the Dutch Experiment on Animals Act and were approved by the local animal welfare committee of the Academic Medical Center.

### Bacteria

*S. pneumoniae* serotype 2 strain D39 and serotype 3 strain WU2 (a kind gift from Dr. D.E. Briles, University of Alabama at Birmingham, AL) were grown to a mid-logarithmic phase at 37°C in Todd-Hewitt broth enriched with 0.5% yeast extract. Bacteria were harvested by centrifugation at 2,900x*g* for 15 min and washed three times in sterile phosphate buffered saline (PBS). Bacteria were diluted to appropriate concentrations to induce infection or heat killed at 70°C for 30 minutes for ex vivo stimulations. Fluorescein-isothiocyanate (FITC) labeling of heat killed bacteria was done by incubation with 0.2 mg/ml FITC (Sigma-Aldrich) in 0.1 M NaHCO_3_, pH 9.0 for 30 minutes at 37°C. After washing the FITC labelled heat killed bacteria were suspended in PBS.

### Experimental Study Design

All animals were between 8 and 14 weeks of age during the experiments; experimental groups were age-matched. Sepsis was induced by intravenous injection with ~400 colony forming units (CFU) D39 or WU2 in 100 µL PBS into the tail vein. Pneumonia-associated sepsis was induced by intranasal inoculation with ~2x10^6^ CFU D39 or ~4x10^3^ CFU WU2 in 50 µL PBS as previously described (31–33). Lung inflammation was induced by intranasal instillation of 100 µg lipoteichoic acid (LTA) (Invivogen) as previously described (34). Mice were sacrificed at predefined time points and samples were harvested and processed as described previously (31–33). Survival was monitored in separate experiments.

For *in vivo* alveolar macrophage phagocytosis, mice were intranasally inoculated with 50 µL PBS containing 5x10^6^ CFU FITC-labelled *S. pneumoniae* D39 and sacrificed after 4 hours. The lung was lavaged as previously described (31). Cells in bronchoalveolar lavage fluid (BALF) were analyzed by flow cytometry as described below.

### *Ex Vivo* Assays

Naive mice were sacrificed for *ex vivo* analysis as described previously (31). Alveolar macrophages were harvested as described (31) and plated at a concentration of 25,000 cells in 200 ul per well in 96-wells F bottom cell culture plates in Roswell Park Memorial Institute medium 1640 (RPMI; ThermoFisher) supplemented with 10% fetal calf serum (FCS) (Hycult), 2 mM L-glutamine (Sigma-Aldrich) and 0.1 units/mL penicillin and streptomycin (Sigma-Aldrich). Before stimulation cells were washed with RPMI1640 complete medium and stimulated with heat killed *S. pneumoniae* D39 (1000:1 bacterium:cell ratio thus 1.25x10^8^ CFU/mL) in medium for 24 hours after which supernatant was collected and stored at -20°C until further analysis.

Blood was collected in heparin tubes (BD Biosciences) for whole blood experiments. For neutrophil activation, whole blood was plated in U bottom wells and incubated with heat killed *S. pneumoniae* D39 in medium at a final concentration of 1x10^7^ CFU/mL at 37 °C for 1 or 2 hours. For neutrophil phagocytosis, whole blood was plated in U bottom wells and incubated with 5x10^6^ CFU FITC-labeled *S. pneumoniae* D39 per 50 ul of blood for 60 minutes at either 4 or 37°C. After incubations, erythrocytes were lysed and leukocytes were analyzed by flow cytometry.

Single cell suspensions of the lung were generated by incubation with 1 mg/ml collagenase D (Roche) for 30 minutes at 37°C followed by passage through a 19G needle. Single cell suspensions of the spleen were generated by passage through a 100 µm mesh.

### Flow Cytometry

Prior to flow cytometry, total cell counts of BALF, blood and spleen were determined with a Coulter cell counter (Beckman Coulter). Cells subjected to flow cytometry were washed and resuspended in FACS buffer (PBS containing 5% BSA, 0.35 mM EDTA, 0.01% NaN_3_). Cell staining was performed according to manufacturer’s recommendations. A table containing the antibodies used is provided in the supplemental text (**Table S2**). Flow cytometry was performed using a FACSCANTO II (BD Biosciences) or Cytoflex S (Beckman Coulter) and data were analyzed using FlowJo software (Tree Star, Ashland, OR). For cell sorting, samples were sorted using a BD INFLUX (BD Biosciences). For neutrophil whole blood phagocytosis, extracellular FITC was quenched with 0.4% trypan blue solution (ThermoFisher) and the phagocytic index was calculated with the formula: (mean fluorescence of the phagocytic cell fraction at 37°C x % positive cells at 37°C) - (mean fluorescence of the phagocytic cell fraction at 4°C x % positive cells at 4°C) (35). For *in vivo* alveolar macrophage phagocytosis, the phagocytic index was calculated with the formula: (mean fluorescence of the phagocytic cell fraction at 37°C x % positive cells at 37°C).

Gating strategies for lung, blood and spleen cell subsets are shown in **Figures S1, S2**. Gating strategies for BALF (34) and neutrophil maturity (22) were done as shown previously.

### Western Blot of Sorted Cells

Cells were sorted into IMDM medium supplemented with 10% fetal calf serum (FCS) (Hycult), 2 mM L-glutamine (Sigma-Aldrich) and 0.1 units/mL penicillin-streptomycin (Sigma-Aldrich). Cells were washed and lysed in lysis buffer (50 mM Tris HCl, 150 mM NaCl, pH 8.0, 1% Triton-X100). The lysate was centrifuged at 14000*g* and supernatant was collected. Western blot analysis was performed as described previously (34). Blots were incubated with rabbit anti-Btk (clone D3H5; Cell Signaling Technology) and rabbit anti-β-actin (Cell Signaling Technology).

### Histopathology and Immunohistochemistry

Four micrometer lung sections were stained with hematoxylin and eosin. Lung inflammation (interstitial inflammation, endothelialitis, bronchitis, oedema, pleuritis) and damage was scored by a pathologist blinded for group identity as previously described (31–33). For analysis of neutrophil influx in the lung and Kupffer cell numbers in the liver sections were stained with rat anti-mouse Ly-6G FITC (1A8)(BioLegend) and F4/80 FITC (BM8) (eBioscience), respectively, as previously described (31–33). Immunohistochemical stainings were quantified by digital image analysis. Slides were scanned with the Philips IntelliSight Ultra FastScanner 1.6RA (Philips Digital Pathology Solutions) and the amount of immune-positivity was measured as percentage of the total lung surface using Image-Pro Premier (Media Cybernatics). Immunohistochemical double staining was performed as previously described (28). For immunofluorescence analysis, spleen material was fixed in 1% paraformaldehyde and cryoprotected in 30% sucrose. Five μm cryosections were stained for red pulp macrophages (F4/80^+^ cells), marginal zone macrophages (SIGNR1^+^ cells), marginal metallophilic macrophages (CD169^+^ cells) and Btk using F4/80-AF700 (BM8), SIGNR1-A647 (22D1), CD169-eF660 (SER4) (all eBioscience) and Btk-A488 (D3H5; Cell Signaling Technology). Nuclei were stained with DAPI or Hoechst 33342 (ThermoFisher). Specificity of Btk-AF488 in immunofluorescence analysis of Btk was previously demonstrated (28). Confocal microscopy was performed using a Leica TCS SP8 DLS microscope and Leica Application Suite X software.

### ELISA and Other Assays

Myeloperoxidase (MPO), neutrophil gelatinase-associated lipocalin (NGAL), matrix metallopeptidase 9 (MMP9), C-X-C motif ligand (CXCL)1, CXCL2, interleukin (IL)-6, IL-1β and tumor necrosis factor (TNF) were measured by ELISA (all R&D Systems) or mouse inflammation kit Cytometric Beads Array (BD Biosciences). For measurement of plasma natural antibodies levels, plates were coated with 10^8^ CFU heat-killed *S. pneumoniae* D39 or WU2, 5 µg/ml capsular polysaccharides from serotype 2 or serotype 3 pneumococci (ATCC) or 10 µg/ml phosphocholine-BSA (Biosearch Technologies) and bound antibodies were detected using HRP-conjugated anti-mouse-IgM or -IgG3 antibodies (both from Southern Biotechnology Associates).

### Statistical Analysis

Data are expressed as box- and whisker plots showing the smallest value, lower quartile, median, upper quartile and largest value. Data were analyzed using GraphPad Prism software. Since most of the data presented were not normally distributed as determined by Kolmogorov-Smirnov test or had low sample size, non-parametric tests were used. Kruskall-Wallis test was used for comparison of three groups and Mann-Whitney U test for comparison between two groups. To correct for multiple comparisons, the P-value was adjusted for a false discovery rate using the Benjamini and Hochberg method. Survival curves are depicted as Kaplan–Meier plots and were compared using the log-rank test. A P-value below 0.05 was considered statistically significant.

## Results

### Anti-Pneumococcal Natural Antibodies Are Minimally Protective During Pneumosepsis

To elucidate the role of Btk in B cells and non-B cells in innate host defense against *S. pneumoniae* in a physiological setting, we employed WT, Btk^-/-^ and Btk^-/-^ mice with transgenic Btk expression in B cells (Cd19-Btk^+^ mice) (26, 36). Male mice were used for these studies, since XLA is observed mostly in male human beings. In Cd19-Btk^+^ mice, Btk protein was detected in B cells at slightly higher level as compared to WT mice [as reported (26)], but not in alveolar macrophages, lung dendritic cells, neutrophils or monocytes (**Figure S3A**). In Cd19-Btk^+^ mice, all functional and developmental B cell defects observed in Btk^-/-^ mice are restored (26, 36). We subjected WT, Btk^-/-^ and CD19-Btk^+^ mice to pneumonia and pneumonia-derived sepsis by intranasal inoculation with the serotype 2 strain D39. Analysis of plasma for natural IgM and IgG_3_ antibody levels against pneumococcal antigens including phosphocholine, serotype 2 capsular polysaccharide and whole D39 pneumococci showed that natural antibody levels are restored in Cd19-Btk^+^ mice (**Table S3**).

**Figure 1:**
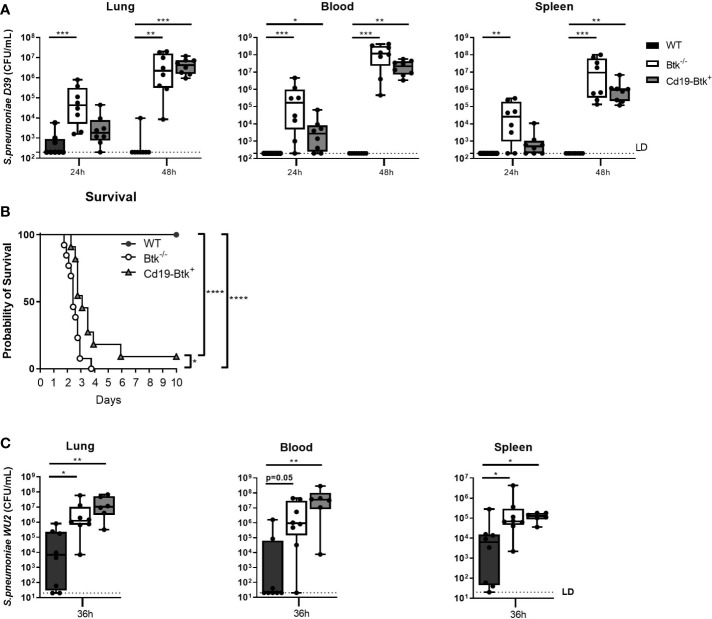
Anti-pneumococcal natural antibodies improve host defense against D39 and WU2 during pneumosepsis to a limited extent. **(A)** WT, Btk^-/-^ and Cd19-Btk^+^ mice were intranasally inoculated with 2x10^6^ CFU D39 (n=8 per group) and sacrificed 24 or 48 hours later. Bacterial counts in lung, blood and spleen are displayed. **(B)** Kaplan-Meier curve of the survival of WT (n=13), Btk^-/-^ (n=13) or Cd19-Btk^+^ (n=11) mice after intranasal inoculation with D39. **(C)** WT, Btk^-/-^ and Cd19-Btk^+^ mice were intranasally inoculated with 4000 CFU WU2 (n=6-8 per group) and sacrificed 36 hours later. Bacterial counts (CFU/mL) in lung, blood and spleen are displayed. For **(A, B)** data are expressed as box- and whisker plots showing the smallest value, lower quartile, median, upper quartile and largest value. LD= limit of detection. *p < 0.05, **p < 0.01, ***p < 0.001, ****p < 0.0001, Kruskall-Wallis test with false discovery rate correction **(A, C)** and log-rank test **(B)**.

Upon intranasal infection with D39, Btk^-/-^ mice as well as Cd19-Btk^+^ mice displayed significantly higher bacterial loads compared to WT mice (**Figure 1A**). Early on, at 24 hours of infection, reinforced expression of Btk in B cells provided some protection against *S. pneumoniae* growth, although CFU’s in lung and spleen of Cd19-Btk^+^ mice were not significantly reduced as compared to Btk^-/-^ mice; however, at 48 hours the pneumococcal infection had dramatically, and equally, progressed in lungs, blood and spleen of both Btk^-/-^ and Cd19-Btk^+^ mice (p<0.001 versus WT).

To determine whether restoration of anti-pneumococcal natural antibodies improved survival of Btk^-/-^ mice from pneumococcal pneumosepsis we performed an observational study (**Figure 1B**). All WT mice survived D39 pneumosepsis over a period of 10 days, whereas all Btk^-/-^ mice and 91% of Cd19-Btk^+^ mice died as a result of the infection (both p<0.0001 *vs* WT). Cd19-Btk^+^ mice showed a minor survival advantage over Btk^-/-^ mice (p<0.05). These results indicate that natural antibodies, which are lacking in in Btk^-/-^ mice and restored in Cd19-Btk^+^ mice, are only transiently and partially protective during pneumococcal pneumosepsis with D39.

To investigate whether the observed transient protective effect of natural antibodies during pneumococcal pneumosepsis was strain-specific, we repeated our experiments with pneumococcal strain WU2 (a serotype 3 strain), which was previously shown to be sensitive to natural antibody-mediated antibacterial defense in Xid mice after intravenous injection (14). Prior to infection, we confirmed that Cd19-Btk^+^ mice have restored levels of natural antibodies against serotype 3 capsular polysaccharides and whole WU2 bacteria (**Table S3**). Since WU2 is highly virulent in Xid mice (14), mice were inoculated with a low inoculum and sacrificed 36 hours later. After intranasal inoculation with WU2, we found a similar pattern of impaired bacterial clearance in Cd19-Btk^+^ mice as with D39 (**Figure 1C**); bacterial loads in lung, blood and spleen of Cd19-Btk^+^ mice at t=36h were significantly higher compared to bacterial loads in WT mice (all p<0.05) and did not significantly differ from bacterial loads in Btk^-/-^ mice.

Taken together, these results indicate that anti-pneumococcal natural antibodies are protective only to a limited extent during pneumococcal pneumonia-derived sepsis in mice.

### Anti-Pneumococcal Natural Antibodies Confer Protection Against Blood Borne Infection With Pneumococcal Strain WU2, but Not D39

The unexpected result that natural antibodies contributed only partially to antibacterial defense after infection with pneumococci *via* the airways led us to assess whether anti-pneumococcal natural antibodies contribute to the antibacterial defense during blood borne pneumococcal infections in Btk^-/-^ mice, as shown previously in Xid mice (10, 14). Therefore, we intravenously injected WT, Btk^-/-^ and Cd19-Btk^+^ mice with D39 or WU2 and analyzed bacterial loads in blood, spleen and liver after 24 hours.

**Figure 2:**
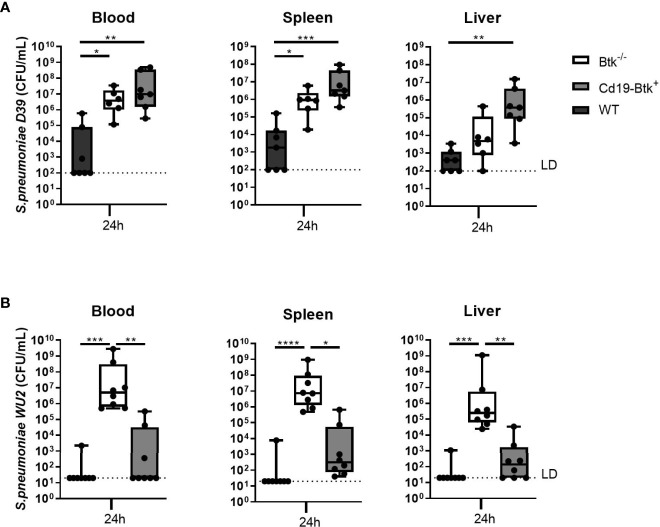
Anti-pneumococcal natural antibodies confer protection against blood borne infection with pneumococcal strain WU2, but not with strain D39. **(A)** WT, Btk^-/-^ and Cd19-Btk^+^ mice were intravenously injected with 400 CFU D39 (n= 6-7 per group) or **(B)** 400 CFU WU2 (n=8). Mice were sacrificed 24 hours later and the bacterial loads in blood, spleen and liver were determined. Data are expressed as box- and whisker plots showing the smallest value, lower quartile, median, upper quartile and largest value. LD= limit of detection. *p < 0.05, **p < 0.01, ***p < 0.001, ****p < 0.0001, Kruskall-Wallis test with false discovery rate correction.

After intravenous injection of D39, bacterial loads in blood and spleen were significantly increased in Btk^-/-^ mice as compared to WT mice (P<0.05 for all comparisons) (**Figure 2A**). In Cd19-Btk^+^ mice, CFU in blood (p<0.01), spleen and liver (p<0.01) were also significantly higher as compared to WT mice and similar to Btk^-/-^ mice. To assess whether anti-pneumococcal antibodies were still present in the circulation of D39-infected Cd19-Btk^+^ mice, we analyzed D39-specific IgM and IgG3 levels in plasma of infected mice. D39-specific IgM and IgG3 levels in plasma of infected Cd19-Btk mice were comparable to the levels in infected WT mice (**Table S4**), in which blood borne infection with D39 was almost completely cleared.

After inoculation of WU2 (**Figure 2B**), bacterial loads in blood, spleen and liver were significantly increased in Btk^-/-^ mice as compared to WT mice (p<0.001 for all comparisons). In Cd19-Btk^+^ mice, CFU in blood, spleen and liver were significantly reduced as compared to Btk^-/-^ mice (p<0.05 for all comparisons) and not significantly different from WT mice. WU2-specific IgM and IgG3 levels in plasma of infected Cd19-Btk^+^ mice were comparable to the levels in infected WT mice (**Table S4**).

These results reveal that natural antibodies are able to protect mice from blood borne infection caused by WU2, but not D39 pneumococci. Taken together, the results from infections with D39 and WU2 *via* the intranasal or intravenous route indicate that natural antibodies do not unequivocally contribute to antibacterial defense against *S. pneumoniae* and that particularly after infection *via* the airways (the more clinically relevant route) do not confer complete protection. These findings reveal that susceptibility of Btk^-/-^ mice for *S. pneumoniae* infection may result also from a critical role of Btk in cells other than B cells. In further experiments we used the pneumonia-derived sepsis model with D39 to test this hypothesis.

### Btk Expression in MhcII^+^ Cells Contributes to Host Defense During Pneumococcal Pneumosepsis

To investigate the role of Btk in myeloid cells during innate host defense against pneumococci, we initially made use of MhcII-Btk^+^ mice (27) which express Btk under control of the Mhc class II promoter in a Btk^-/-^ background. In MhcII-Btk^+^ mice, Btk protein was detected in B cells at slightly higher level as compared to WT mice (as found before (27)). Moreover, in this strain pulmonary and splenic dendritic cells, and to a lesser extent alveolar macrophages, Ly-6C- and Ly-6C+ monocytes and Kupffer cells also express Btk, whereas neutrophils and splenic macrophages do not express Btk (**Figures S3A–C, S4A–D**). Similar to Cd19-Btk^+^ mice, MhcII-Btk^+^ mice showed restored anti-pneumococcal natural antibody levels (**Table S3**), in accordance with Btk expression in B cells (**Figure S3A**).

**Figure 3:**
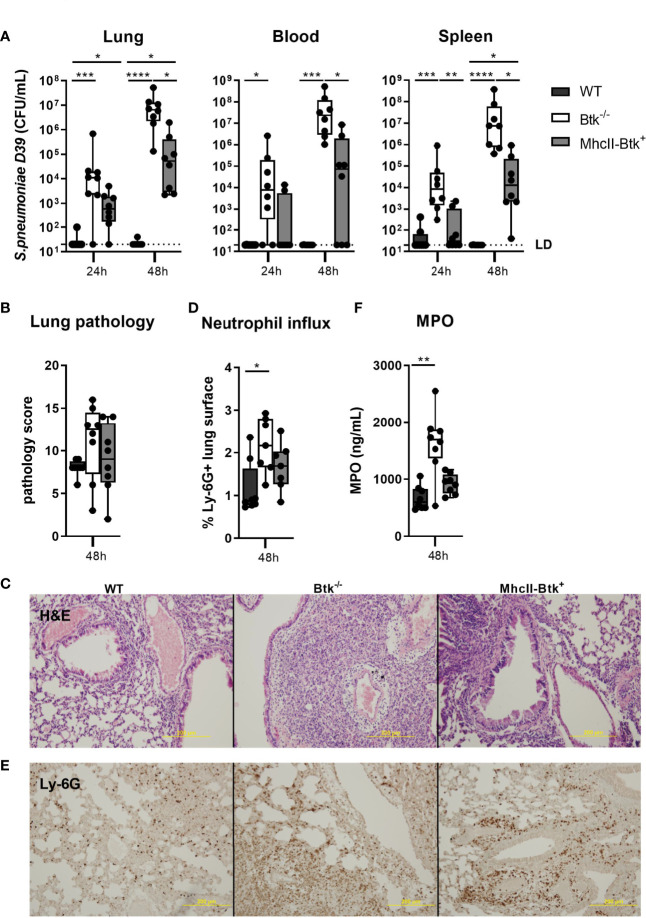
Transgenic Btk expression in MhcII^+^ cells of Btk^-/-^ mice improves host defense against D39 pneumococci. WT, Btk^-/-^ or MhcII-Btk^+^ (n=8 per group) mice were intranasally inoculated with 2x10^6^ CFU D39 and sacrificed after 24 or 48 hours. **(A)** Bacterial counts in the lung, blood and spleen. **(B, C)** Pathology score and representative hematoxylin and eosin (H&E) pictures at 48 hours. **(D, E)** Percentage of Ly-6G positive lung surface and representative pictures of Ly-6G staining at 48 hours. **(F)** Concentrations of MPO in lung homogenates at 48 hours. Data are expressed as box- and whisker plots showing the smallest value, lower quartile, median, upper quartile and largest value. LD= limit of detection. *p < 0.05, **p < 0.01, ***p < 0.001, ****p < 0.0001, Kruskall-Wallis test with false discovery rate correction.

Upon intranasal inoculation with D39 pneumococci, MhcII-Btk^+^ mice showed increased resistance to bacterial outgrowth as compared to Btk^-/-^ mice but not to the same extent as in WT mice (**Figure 3A**). Bacterial loads in lungs and spleen of MhcII-Btk^+^ mice were significantly higher compared to those in WT mice (both p<0.05) but lower compared to loads in Btk^-/-^ mice (lung, blood and spleen, p<0.05).

To determine the role of Btk in MhcII+ cells in the regulation of lung inflammation induced by *S. pneumoniae*, we assessed lung pathology, the extent of neutrophil influx into the lung, and levels of inflammatory cytokines in the lungs of MhcII-Btk^+^, Btk^-/-^ and WT mice at 48 hours after intranasal inoculation. Despite higher bacterial loads in lungs of Btk^-/-^ and MhcII-Btk^+^ mice compared to WT mice, lung pathology was not exaggerated in Btk^-/-^ or MhcII-Btk^+^ mice (**Figures 3B, C**). Neutrophil influx, as reflected by Ly6G staining in lung sections and total lung MPO levels (**Figures 3D–F**), and TNF, IL-1β and IL-6 levels (**Table S5**) in the lung of WT, Btk^-/-^ and MhcII-Btk^+^ mice paralleled bacterial loads in the lung.

To obtain further insight in the enhanced host defense against D39 pneumococci of MhcII-Btk^+^ mice compared to Btk^-/-^ mice, we analyzed subsets of innate immune cells in lung, blood, spleen and liver harvested from naïve mice. Alveolar macrophage and lung dendritic cells numbers were similar in WT, Btk^-/-^ and MhcII-Btk^+^ mice (**Figure S5A**). MhcII-driven Btk expression also did not effect on numbers of liver and spleen macrophages (**Figures S5B, S6**), spleen B-, T- lymphocyte and neutrophils (**Figures S5C, D**). In contrast, MhcII-driven Btk expression caused elevated monocyte numbers in blood (both p<0.01; **Figure S5E**) and increased splenic CD4^-^CD8^-^ dendritic cells (p<0.05; **Figure S5F**). Furthermore, B cell numbers in blood of MhcII-Btk^+^ mice were reduced as compared to WT mice (p<0.01), and comparable to B cell numbers in Btk^-/-^ mice (**Figure S5G**).

Taken together, these data indicate that MhcII-driven expression of Btk confers partial protection against D39, which may result from enhanced innate immunity by myeloid cells.

### Btk Contributes to Alveolar Macrophage Responsiveness to Pneumococci but Lysmcre- Btk^fl^/Y Mice Have an Unaltered Antibacterial Defense During Pneumococcal Pneumosepsis

**Figure 4:**
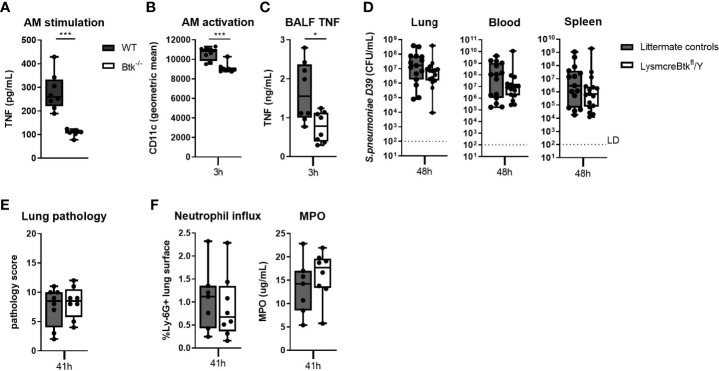
Btk contributes to alveolar macrophage responsiveness to pneumococci but LysM driven deficiency of Btk does not influence resistance against bacterial outgrowth during pneumococcal pneumosepsis. **(A)** TNF levels in supernatant of WT and Btk^-/-^ alveolar macrophages (AM) stimulated for 24 hours with heat killed D39 ex vivo (n=8 per group). **(B–E)** LysmcreBtk^fl^/Y mice and littermate controls were intranasally inoculated with 2x10^6^ CFU D39 and sacrificed after 3 and 48 hours (n=15 per groups). **(B)** Alveolar macrophage CD11c expression (geometric mean) and **(C)** BALF TNF levels 3 hours after induction of infection. **(D)** Bacterial loads in the lung, blood and spleen 48 hours after induction of infection. **(E)** Pathology score, **(F)** percentage of Ly-6G positive lung surface and concentrations MPO in lung homogenate 48 hours after induction of infection. Data are expressed as box- and whisker plots showing the smallest value, lower quartile, median, upper quartile and largest value. LD= limit of detection. *p < 0.05, ***p < 0.001, Mann-Whitney U test.

Alveolar macrophages play an important role in host defense against pneumococci (37–39). To establish whether Btk is required for alveolar macrophage effector functions, we analyzed the responsiveness of WT and Btk-deficient alveolar macrophages to pneumococci *in vitro* and *in vivo*. Alveolar macrophages isolated from Btk^-/-^ mice secreted significantly less TNF (p<0.001) (**Figure 4A**), CXCL1 (p<0.01), CXCL2 (p<0.05) and IL-6 (p<0.001) (**Table S6**) in response to D39 as compared to alveolar macrophages from WT mice but were not hampered in phagocytosis of D39 (**Figure S7**).

To determine whether Btk also contributes to alveolar macrophage responsiveness to pneumococci *in vivo*, we generated Lysmcre-Btk^fl^/Y mice. Analysis of cell specific Btk expression in Lysmcre-Btk^fl^/Y mice revealed that besides alveolar macrophages, also the majority of splenic macrophages and Kupffer cells lacked Btk expression (**Figures S8A, B**, **S9A–D**). Lysmcre-Btk^fl^/Y mice also showed significantly diminished Btk expression in lung dendritic cells, neutrophils and Ly-6C^-^ monocytes (**Figure S8A**) but not in splenic dendritic cells and Ly6C^+^ monocytes (**Figure S8C**) (40, 41). Analysis of the percentage of alveolar macrophages, lung dendritic cells, blood monocytes, neutrophils, B and T lymphocytes, liver Kupffer cells and number of splenic neutrophils, dendritic cells and B and T lymphocytes showed no alteration in Lysmcre-Btk^fl^/Y mice as compared to littermate controls (**Figures S8D–J**,**S10**). Moreover, natural anti-pneumococcal antibody levels were comparable in Lysmcre-Btk^fl^/Y and littermate control mice (**Table S7**). We analyzed alveolar macrophage activation and inflammatory cytokine release in in BALF of Lysmcre-Btk^fl^/Y mice 3 hours after D39 inoculation. Lung CFU in Lysmcre-Btk^fl^/Y mice and littermate controls were similar at 3 hours after inoculation (data not shown). Alveolar macrophage activation, measured by CD11c expression (34), was significantly decreased in Lysmcre-Btk^fl^/Y mice (p<0.001) although to a small extent (**Figure 4B**). Also, TNF levels in BALF were significantly lower in Lysmcre-Btk^fl^/Y mice as compared to controls (p<0.05) 3 hours after D39 infection (**Figure 4C**), whereas IL-6, CXCL1 and CXCL2 levels were not altered (**Table S8**).

Next, we analyzed the impact of reduced Btk expression in specific myeloid cells on bacterial outgrowth and inflammatory parameters at 48 hours after intranasal inoculation of D39. Lysmcre-Btk^fl^/Y mice showed no difference in bacterial loads in lung, blood and spleen compared to littermate controls (**Figure 4D**). Lung pathology (**Figure 4E**) and neutrophil influx in the lung (**Figure 4F**), measured by Ly-6G staining and MPO, were not different between Lysmcre-Btk^fl^/Y and controls. TNF levels in lung homogenates were significantly lower in Lysmcre-Btk^fl^/Y mice compared to controls (p<0.05), whereas IL-6 and IL-1β levels were not different between groups (**Table S9**).

Taken together, these results indicate that Btk in alveolar macrophages contributes to the early inflammatory response in the lung induced by pneumococcal infection, but is not essential for host defense during pneumococcal pneumosepsis at later time points.

### Mrp8cre-Btk^fl^/Y Mice Show an Impaired Antibacterial Defense During Pneumococcal Pneumosepsis

Considering the incomplete deletion of Btk in neutrophils of Lysmcre-Btk^fl^/Y mice (**Figure S8A**), we decided to investigate the contribution of neutrophil Btk in the host response during pneumococcal pneumonia in more detail. To this end we generated neutrophil specific Btk deficient (Mrp8cre-Btk^fl^/Y) mice. Previous studies have indicated that higher percentages of neutrophils are depleted from floxed target proteins in Mrp8cre mice compared to Lysmcre mice (42, 43). Western blot analysis of sorted cells from Mrp8cre-Btk^fl^/Y and control mice showed a markedly decreased expression of Btk in neutrophils, but not in alveolar macrophages, Ly-6C^+^ or Ly-6C^-^ monocytes or B cells (**Figure S11A**). Reduced expression of Btk in neutrophils from Mrp8cre-Btk^fl^/Y did not impact neutrophil numbers in blood and spleen, nor the number of other cell types (**Figures S11B–F**). As expected, reduced expression of Btk in neutrophils from Mrp8cre-Btk^fl^/Y also did not affect natural anti-pneumococcal antibody levels (**Table S10**).

**Figure 5:**
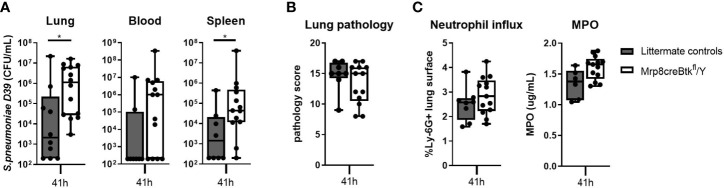
Neutrophil specific Btk expression is essential for host defense against D39 pneumococci. **(A)** Bacterial loads in lung, blood and spleen of Mrp8creBtk^fl^/Y (n=10) mice and littermate controls (n=14) 41 hours after intranasal inoculated with D39. **(B)** Pathology score. **(C)** Percentage of Ly-6G positive lung surface and concentrations MPO in lung homogenate. Data are expressed as box- and whisker plots showing the smallest value, lower quartile, median, upper quartile and largest value. *p < 0.05, Mann-Whitney U test.

To determine the contribution of Btk expression in neutrophils to host defense against *S. pneumoniae*-evoked pneumosepsis, we inoculated Mrp8cre-Btk^fl^/Y mice and littermate controls with D39 pneumococci *via* the airways and sacrificed all mice 41 hours later. Bacterial loads in lungs and spleen were significantly higher in Mrp8cre-Btk^fl^/Y mice (both p<0.05)(**Figure 5A**) and a similar trend was observed for bacterial loads in blood. To determine whether the increased bacterial loads in spleen and blood resulted from impaired systemic host defense, we intravenously injected Mrp8cre-Btk^fl^/Y mice with D39 streptococci and assessed bacterial loads 24 hours later. Mrp8cre-Btk^fl^/Y mice showed similar bacterial loads in blood, spleen and liver after intravenous injection with D39 pneumococci as littermate controls (**Figure S12**), indicating that the impaired host defense in Mrp8cre-Btk^fl^/Y mice after intranasal D39 inoculation is due to defects in pulmonary neutrophil function.

Pulmonary inflammation was not altered in Mrp8cre-Btk^fl^/Y mice during pneumococcal pneumonia as compared with control mice, as reflected by similar lung pathology scores (**Figure 5B**), neutrophil influx (Ly-6G staining and MPO levels in homogenates)(**Figure 5C**) and TNF and IL-1β levels in the lung (**Table S11**); only lung IL-6 levels were increased in Mrp8cre-Btk^fl^/Y mice as compared to controls (p<0.05) (**Table S11**).

These results indicate that Btk in neutrophils is essential for the antibacterial response during pneumococcal pneumonia and suggest that impaired host defense of Btk^-/-^ mice against *S. pneumoniae* infection may result from Btk deficiency in neutrophils.

### Btk Deficient Neutrophils Show Reduced Effector Functions in Response to LTA and Pneumococci

**Figure 6:**
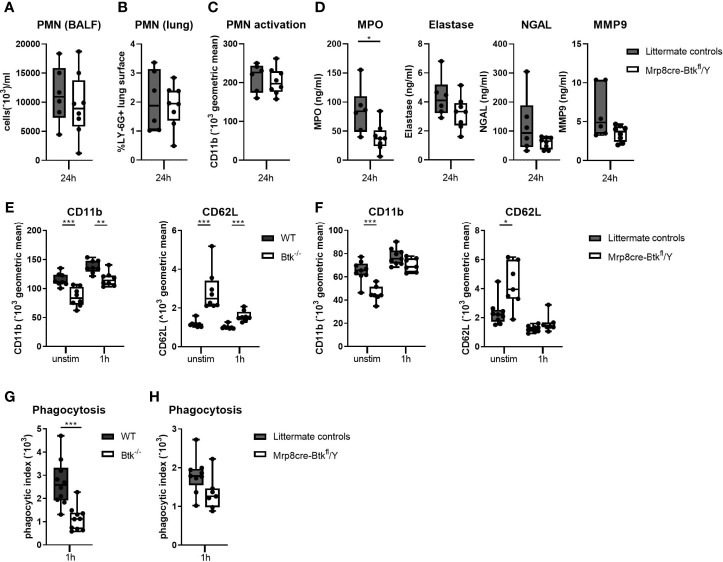
Btk deficient neutrophils show reduced effector functions in response to LTA and pneumococci. **(A-D)** Mrp8cre-Btk^fl^/Y (n=6) and littermate controls (n=8) were sacrificed 24 hours after intranasal administration of lipoteichoic acid (LTA). **(A)** Numbers neutrophils (PMN) in BALF. **(B)** Percentage of LY-6G positive lung surface. **(C)** CD11b expression (geometric mean) on BALF PMN. **(D)** BALF concentrations of the PMN degranulation products myeloperoxidase (MPO), elastase, neutrophil gelatinase-associated lipocalin (NGAL) and matrix metallopeptidase 9 (MMP9). **(E, F)** CD11b and CD62L surface expression on blood neutrophils from **(E)** WT and Btk^-/-^ (n=8 per group) and **(F)** littermate control and Mrp8creBtk^fl^/Y (n=7-9 per group) at baseline or after stimulation for 1 hour with D39. **(G, H)** Whole blood of **(G)** WT and Btk^-/-^ mice (n=10 per group) or **(H)** littermate control and Mrp8creBtk^fl^/Y mice (n7-9 per group) was incubated for 60 minutes with FITC-labelled D39 after which the neutrophil phagocytic index of was determined. Data are expressed as box- and whisker plots showing the smallest value, lower quartile, median, upper quartile and largest value. *p < 0.05, **p < 0.01, ***p < 0.001, Mann-Whitney U test.

Since pulmonary inflammatory responses during D39-induced pneumonia are dependent on the bacterial loads (**Figure 3** and **Table S5**), the impact of neutrophil Btk depletion on pulmonary neutrophil influx and degranulation might be concealed by differences in bacterial numbers between Mrp8cre-Btk^fl^/Y and control mice. Therefore, we investigated the impact of Btk deficiency on neutrophil migration, activation and degranulation in a model of acute lung inflammation elicited by local administration of LTA, a stable and robust challenge with relevance for pneumococcal infection (34). Mrp8cre-Btk^fl^/Y and control mice were administered LTA *via* the airways after which lung and BALF were harvested 24 hours later. Neutrophil influx in the lung (as determined by BALF neutrophil numbers and by percentage of LY-6G positive lung surface) were not different between groups (**Figures 6A, B**), suggesting that neutrophil Btk is not required for neutrophil migration into the lung. Neutrophil activation, measured as CD11b expression, was also not different between groups (**Figure 6C**). Analysis of neutrophil degranulation products in BALF showed lower levels of MPO, elastase, NGAL and MMP9 in Mrp8cre-Btk^fl^/Y mice as compared to littermate controls, which reached significance only for MPO (p<0.05) (**Figure 6D**).

To obtain further insight in the role of Btk in neutrophil degranulation, we analyzed CD11b expression on blood neutrophils from naïve Btk^-/-^ and WT mice *ex vivo*. Expression of CD11b is upregulated through degranulation (44). Unstimulated Btk^-/-^ neutrophils showed significantly decreased surface CD11b expression as compared to control neutrophils (p<0.001) (**Figure 6E** and **Figure S13**) and the differences in CD11b expression remained upon stimulation with D39 pneumococci (p<0.01). Concurrent analysis of CD62L, an adhesion molecule that is constitutively expressed at high levels on neutrophils and shed following activation (45), showed an inversed expression pattern as compared to CD11b on neutrophils from WT and Btk^-/-^ mice (**Figure 6E** and **Figure S13**). Similarly, unstimulated neutrophils from Mrp8cre-Btk^fl^/Y mice showed significantly decreased surface CD11b expression and increased CD62L expression as compared to unstimulated littermate controls (p<0.001, **Figure 6F** and **Figure S13**). Stimulation with D39 pneumococci abolished the difference in CD11b and CD62L expression on neutrophils between these groups.

Furthermore, we determined whether Btk in neutrophils is required for phagocytosis of D39 pneumococci. Blood neutrophils from Btk^-/-^ mice displayed a reduced capacity to phagocytose D39 (p<0.001, **Figure 6G** and **Figure S13**). Experiments with neutrophils from Mrp8cre-Btkfl/Y mice corroborated these findings, although this did not reach significance (p=0.07, **Figure 6H** and **Figure S13**).

Taken together, these results indicate that Btk-deficient neutrophils are less primed as compared to WT neutrophils and display diminished effector functions against pneumococci.

## Discussion

XLA patients and Btk deficient mice are highly susceptible to infections (12), especially with pneumococci (8, 10). Since the introduction of immunoglobulin replacement therapy for XLA, infection rates have dropped dramatically and most treated patients survive into adulthood (9, 13). In accordance, Btk^-/-^ mice inoculated with pneumococci in the presence of natural IgM or IgG3 antibodies, were capable of clearing the infection (10, 14). These observations have led to the dogma that Btk in myeloid cells - although abundantly expressed herein - is dispensable for adequate innate immune responses against pneumococci. However, despite immunoglobulin treatment, a considerable number of XLA patients still experience recurrent respiratory tract infections (8, 9, 24). Therefore, in the present study, we further investigated the role of Btk in B cells and myeloid cells in the antibacterial defense against pneumococci. The results of our study show that Btk^-/-^ mice with reinforced expression of Btk in B cells, which restored their natural anti-pneumococcal IgM and IgG3 levels, were only minimally protected against pulmonary pneumococcal infection, indicating a role for Btk in cells other than B cells. Btk^-/-^ mice with reinforced Btk expression in MhcII^+^ cells, including B cells, dendritic cells and macrophages, did show improved antibacterial defense as compared to Btk^-/-^ mice, suggesting that Btk in myeloid cells contributes to innate host defense against pneumococci. Moreover, Mrp8creBtk^fl^/Y mice, but not LysmcreBtk^fl^/Y mice, displayed decreased antibacterial defense upon pneumococcal-induced pneumosepsis. To our knowledge, we show here for the first time that Btk in myeloid cells and particularly in neutrophils is required for the resistance to *S. pneumoniae* infection.

Natural IgM and IgG_3_ antibodies, constitutively produced by B-1 B cells, are an important component of the innate immune response (46, 47). Natural antibodies recognize diverse microbial antigens and exert various activities including pathogen and toxin neutralization, complement activation and phagocytosis (47, 48). Several pneumococcal strains have been found to be sensitive to natural antibody mediated clearance after blood borne infection (10, 14). However, previous studies (10, 14, 49) showed that certain pneumococcal strains are resistant to antibody mediated clearance after intravenous injection. We here show that intravenously injected *S. pneumoniae* D39 is not cleared in Btk^-/-^ mice with rescued natural antibody production, whereas *S. pneumoniae* WU2 is. Others have shown similar results with mice intravenously injected with D39 or WU2 after passive immunization with antibodies against pneumococcal surface protein A (PspA) (49). Immunized mice injected with WU2 showed a survival advantage compared to non-immunized mice, whereas immunization before D39 injection did not improve survival (49). The resistance of D39 to antibody mediated clearance was not caused by its capsular or PspA serotype and independent of the amount of antibodies or C3 bound to its surface (49). This suggests that D39 possesses factors other than its capsular or PspA serotype, that can impede antibody-mediated protection. This notion is corroborated by the fact that switch of the polysaccharide capsule, an important virulence factor of pneumococci (50), from serotype 2 (D39) to serotype 3 (WU2) did not alter virulence in mice (51). It has been proposed that, among the wide variety of virulence factors expressed by pneumococci (50), subsets of virulence factors may contribute to persistence of bacteria in distinct anatomical sites (mucosa vs. bloodstream) (52). To our knowledge, it is unknown which virulence factors of pneumococci are important for bloodstream infection and might cause the difference in bloodstream infection between D39 and WU2.

It has been reported that restoration of natural antibodies in Xid mice reduces lung bacterial loads during pneumococcal pneumonia, however, long term effects of natural antibody restoration on survival were not evaluated (53). While our results are in accordance, they indicate that natural antibodies only provide limited protection during pneumococcal pneumonia and suggests that their deficiency may not be the sole reason for the severely impaired host defense against pneumococcal pneumosepsis of Btk^-/-^ mice. Further studies with IVIG treatment of Btk^-/-^ mice may prove (or refute) our conclusion on the limited protective effect of natural antibodies during pneumococcal pneumonia, but were regarded outside the scope of this study on the cell-specific role of Btk in host defense during pneumococcal pneumonia. To corroborate the insufficiency of B-cell/antibody-mediated mechanisms in protective immunity, we attempted to generate conditional Btk-deficient mice that lacked Btk solely in B cells by crossing Btk^fl/fl^ mice with Cd19cre mice. Unexpectedly, Cd19cre-Btk^fl^/Y mice displayed unaltered anti-pneumococcal natural antibody levels as compared to littermate controls (**Table S12**). This indicates that B cells were not sufficiently targeted in these conditional Btk deficient mice, probably due to the relatively late expression of Cd19 during B cell development. Studies with mice expressing cre-recombinase early in B cell development, such as Mb1-cre mice, may be better suitable for this approach.

The results of our experiments with MhcII-Btk^+^ and Mrp8cre-Btk^fl^/Y mice unequivocally reveal that Btk expression in myeloid cells contributes to innate immune host defense against *S. pneumoniae*. We initially made use of a gain-of-function approach with expression of Btk driven by the MhcII promoter in mice that are otherwise deficient for Btk. MhcII-Btk^+^ mice were partially protected against D39 infection, i.e., bacterial loads in the lung, blood and spleen were significantly reduced as compared to Btk^-/-^ mice, also at the late time point when natural antibodies were shown to be no longer protective in Cd19-Btk^+^ mice. These findings indicate that expression of Btk in MhcII+ cells contributes to host defense against D39 pneumococci. Detailed analysis of MhcII-Btk^+^ mice revealed that whilst Btk protein expression was present in B cells, monocytes, alveolar macrophages and dendritic cells, Btk levels were reduced in alveolar macrophages and monocytes and increased in dendritic cells and B cells in comparison to WT mice. A limitation of the current study in the experiments with MhcII-Btk^+^ mice is that Btk protein levels in MHCII expressing cells from these mice are different from Btk protein levels in cells from WT mice. Although increased Btk expression in B cells of MhcII-Btk^+^ mice may explain higher anti-pneumococcal antibodies in these mice as compared to WT mice, these increased antibody levels may have had a small effect on host defense of these mice due to their limited protective effect. The reduced bacterial numbers in the lungs of MhcII-Btk^+^ mice as compared to Btk^-/-^ and Cd19-Btk^+^ mice may thus result from reinforced expression of Btk in alveolar macrophages, monocytes and dendritic cells. Strikingly, Lysmcre-Btk^fl^/Y mice, in which alveolar macrophages were completely Btk deficient, did not show impaired resistance to bacterial growth during pneumococcal pneumosepsis, despite a reduced capacity of alveolar macrophages to respond to pneumococci. Alveolar macrophages have been shown to be essential for regulating the innate immune response during *S. pneumoniae* infection (37–39), amongst other by secretion of TNF and therewith restraining bacterial outgrowth (38, 54, 55). Reduced pulmonary TNF production in Lysmcre-Btk^fl^/Y mice, however, did not hamper host defense against pneumococci, suggesting that the reduced TNF levels were sufficient to elicit an adequate immune response. Moreover, reduced expression of Btk in LY-6C^-^ monocytes, dendritic cells and neutrophils of Lysmcre-Btk^fl^/Y mice did also not impact on immunity against *S. pneumoniae*. Although these results seem to be in contradiction with the results from MhcII-Btk^+^ mice, the improved host defense of the latter strain could be explained by over-expression Btk in MHCII^+^ cells, which may alter cellular responses as compared to cells from WT mice, as has been observed in B cells from Cd19-Btk^+^ mice (56). Moreover, increased Btk expression in MHCII+ cells of MhcII-Btk^+^ mice caused a significant increase in the number of monocytes and dendritic cells as compared to WT mice. Since monocytes and dendritic cells may contribute to host defense against pneumococci, these phenotypic changes may explain the enhanced host response of these mice. For this reason, the results obtained with MhcII-Btk^+^ mice should be interpreted with caution. Based upon earlier studies with Lysmcre mice (40, 42, 43) a likely explanation for unaltered bacterial growth in Lysmcre-Btk^fl^/Y mice may be that Btk protein levels in neutrophils, dendritic cells and LY-6C^-^ monocytes were not low enough to influence effector functions. Incomplete deletion of the Btk gene may thus leave sufficient numbers of residual Btk-expressing cells in these myeloid populations to elicit an adequate immune response against pneumococci. In line with this notion, we decided to study the role Btk in neutrophils in more detail using Mrp8cre-Btk^fl^/Y mice. Previously, it was demonstrated that higher proportions of neutrophils are depleted from target proteins in Mrp8cre mice as compared to Lysmcre mice (40, 42, 43). The increased bacterial loads in the lungs, blood and spleen of Mrp8cre-Btk^fl^/Y mice compared to control mice during pneumococcal pneumonia indicate that Btk in neutrophils plays an important role in host defense against pneumococci. Neutrophils play a delicate role in host defense against D39-induced pneumonia (57). In this study it was found that 95% reduction of neutrophil numbers (by antibody mediated depletion) resulted in highly increased *S. pneumoniae* loads in lung and blood, but that 77% reduction of neutrophil influx into the lung (by anti-CCL7 treatment) did not affect lung bacterial loads. These findings indicate that D39 induces a robust and redundant neutrophil influx in the lung, but that a fraction of neutrophils is sufficient for adequate pulmonary host defense against D39. In view of the aforementioned studies (42, 43), it is plausible that Btk levels are more reduced in neutrophils from Mrp8cre-Btk^fl^/Y mice as compared to Lysmcre-Btk^fl^/Y mice, and when below a certain threshold hampers neutrophil-mediated host defense against D39. Further studies are required into this matter. The importance of Btk in neutrophil function may also explain why MhcII-Btk^+^ mice, lacking Btk expressing neutrophils, displayed higher bacterial outgrowth as compared to WT mice. Our study shows that neutrophils of Mrp8cre-Btk^fl^/Y mice and Btk^-/-^ mice are impaired in effector functions against pneumococci. Neutrophilic phagocytosis is important for clearance of pneumococci during pneumonia (58), and Btk-deficient neutrophils demonstrated reduced bacterial uptake. Furthermore, our finding that BALF MPO levels in Mrp8cre-Btk^fl^/Y mice after LTA induced lung inflammation were decreased, indicates that Btk deficient neutrophils have a decreased capacity to secrete MPO. It was previously shown that neutrophils from Xid mice have a reduced capacity to produce reactive oxygen species (ROS) (59), and the reduced capacity to release MPO that we report here is consistent with this finding. Although MPO is important for pneumococcal clearance during acute otitis media (60), the contribution of MPO to host defense against pneumococcal pneumonia is less clear (61). Mice deficient for gp91(phox), a component of NADP oxidase important for ROS production, did not show impaired bacterial clearance after pneumococcal challenge (62). In line with these findings, we observed that D39 pneumococci did not induce ROS production in neutrophils *in vitro* (data not shown), suggesting that effect of neutrophils during pneumococcal pneumonia is most probably not due to ROS production but may relate to other effector functions. Our study further shows that naïve blood neutrophils of Btk^-/-^ mice and Mrp8cre-Btk^fl^/Y mice have decreased CD11b and increased CD62L surface expression. However, upon whole blood stimulation with pneumococci, neutrophils from Mrp8cre-Btk^fl^/Y mice were capable to restore expression to littermate levels in contrast to neutrophils from Btk^-/-^ mice. Albeit CD11b and CD62L are important for neutrophil migration, and Btk has been implicated in neutrophil adhesion to and crawling along endothelium and migration into tissue (23, 63, 64), the deviant expression levels of these adhesion molecules in unstimulated cells did not result in decreased neutrophil migration in Mrp8cre-Btk^fl^/Y mice upon pneumococcal pneumonia or LTA induced lung inflammation. It might, however, represent a state of reduced activation in unstimulated conditions (65).

In experiments with Cd19-Btk^+^ and MhcII-Btk^+^ mice, low bacterial loads were found in WT control mice, while in experiments with Lysmcre-Btk^fl^/Y and Mrp8cre-Btk^fl^/Y mice high CFU loads were observed in control mice. Although differences in the composition of microbiota between mouse strains may impact on host defense (66), we previously found that disruption of the gut microbiota by broad spectrum antibiotics had a modest effect on lung D39 loads (67). Since all experiments were performed in a strictly controlled manner, we have no clear explanation for these differences in bacterial loads.

Few studies suggest a role for Btk in effector functions of human neutrophils. Studies with neutrophils from XLA patients demonstrated either that Btk deficiency did not impact on TLR-induced neutrophil activation and oxidative burst (68), or enhanced the production of reactive oxygen species (69). In contrast, recent studies with Btk inhibitors showed that Btk inhibition affected neutrophil function and decreased phagocytosis of fungi, fungi- and FcR-induced oxidative burst, and bacterial killing (70, 71).

Recently, it was found that a specific subset of resident neutrophils in the spleen is important for elimination of pneumococci captured by red pulp macrophages (72). These resident neutrophils phagocytose and kill the bacteria, therewith retaining the infection until the T cell-independent antibody response is initiated. We did not find evidence that Btk in splenic neutrophils is involved in this process, since intravenous injection of pneumococci in Mrp8cre-Btk^fl^/Y mice did not result in altered bacterial numbers in blood, spleen and liver as compared to controls.

The results of the current study indicate that the severely impaired host defense of Btk^-/-^ mice against pneumococci cannot be attributed to dysfunction of particular innate immune cell types targeted in the genetically modified mice used. Rather, the data presented here suggest that the whole immunological defect of Btk deficiency is greater than the sum of its parts. It is plausible that absence of natural antibodies, decreased TNF production by alveolar macrophages, functional defects in phagocytosis and MPO secretion of neutrophils, as well as impaired function of platelets (28) might all contribute to impaired host defense of Btk^-/-^ mice during pneumococcal pneumonia. Moreover, our finding that MhcII-Btk^+^ mice are able to restrain pneumococcal outgrowth as compared to Btk^-/-^ mice, suggests that Btk is also required for bactericidal responses of dendritic cells and monocytes. Experiments with CD11ccre-Btk^fl^/Y mice and CCR2cre-Btk^fl^/Y mice may provide further insight in the role for Btk in these cell types in host defense during pneumococcal pneumonia. Furthermore, the impaired host defense of Btk^-/-^ mice against pneumococci also cannot be attributed to a single pattern recognition receptor, since Btk is involved in signaling cascades of a variety of receptors such as TLR, NLRP3 and TREM-1 (16), which all are important for pneumococcal recognition (73).

For our studies we have used male mice, as XLA affects mostly male human beings. Interestingly, one female with XLA has been reported, resulting from a maternally derived X chromosome that was exclusively inactivated and a paternally derived X chromosome with a mutation in BTK (74). Although not investigated in the present study, female mice with homozygous mutation in Btk are expected to respond similarly as male mice with hemizygous mutation in Btk as these mice have identical phenotypes (7).

The involvement of Btk in various inflammatory responses makes it a potentially interesting target to inhibit during overwhelming inflammation, such as sepsis and COVID-19. Pharmacological inhibition of Btk after onset of inflammation decreased lung inflammation in models of murine influenza (75) and pneumococcal pneumonia (34). The effect of Btk inhibition in patients with COVID-19 also seems beneficial (76) and is being further studied (77). In line with these findings, Xid mice are protected from multi-organ failure caused by polymicrobial sepsis (78). The latter study indicates that, in contrast to the findings reported here, a diminished host response due to absence of functional Btk might be beneficial in certain infectious diseases. Further research in this area is warranted, since treatment of patients with B cell malignancies with Ibrutinib is associated with serious adverse infectious events within several prospective clinical trials (11). In this systematic review, it was determined that infectious complications were common in 56% of patients taking Ibrutinib as single-agent and that 21% of the patients on Ibrutinib developed pneumonia. Unfortunately, this review did not reveal how many of these infections resulted from bacteria, such as *S. pneumoniae*. Nevertheless, these numbers indicate that caution should be taken when treating overwhelming inflammation with Btk inhibitors such as Ibrutinib.

In summary, the results of our study indicate that the severely impaired host defense of Btk^-/-^ mice during pneumococcal pneumosepsis is multifactorial and not solely due to the absence of natural antibodies as previously proposed (10, 14). We demonstrate that Btk expression in myeloid cells, and particularly in neutrophils, is required for antibacterial defense against this pathogen. Although Btk is expressed by similar cell types in mice and man (**Table S1**), Btk-dependent signaling pathways differ between these species as exemplified by the difference in B cell depletion in Btk-deficient/Xid mice and XLA patients (6, 16). Therefore, caution is warranted when extrapolating findings from murine studies on Btk to the human situation. Nevertheless, the impaired myeloid cell mediated antibacterial defense caused by Btk deficiency we report here, may explain the recurrent respiratory tract infections in patients with XLA on IVIG treatment and the increased susceptibility to infections in patients treated with ibrutinib (9, 11). Further studies on the cell-specific role of Btk in host defense against persistent infectious agents are therefore needed.

## Data Availability Statement

The raw data supporting the conclusions of this article will be made available by the authors, without undue reservation.

## Ethics Statement

The animal study was reviewed and approved by Animal welfare committee of the Academic Medical Center.

## Author Contributions

AP, AV, and TP designed the study. RH generated Btk^-/-^, Cd19-Btk^+^ and MhcII-Btk^+^ mice. AP, ZL, AV, and RB acquired all data. AP, SF, OB and JR analyzed all pathology. JH generated methods for confocal microscopy. All authors were involved in interpretation of the data. AP, ZL, and AV drafted the manuscript and all authors reviewed and revised it critically for important intellectual content. All authors contributed to the article and approved the submitted version.

## Funding

AP was funded by a PhD scholarship from the Academic Medical Center, Amsterdam, Netherlands. ZL was funded by the Chinese Scholarship Council (CSC No. 201706170060).

## Conflict of Interest

The authors declare that the research was conducted in the absence of any commercial or financial relationships that could be construed as a potential conflict of interest.

## Publisher’s Note

All claims expressed in this article are solely those of the authors and do not necessarily represent those of their affiliated organizations, or those of the publisher, the editors and the reviewers. Any product that may be evaluated in this article, or claim that may be made by its manufacturer, is not guaranteed or endorsed by the publisher.
