# Supplementary material for: Bruton’s Tyrosine Kinase-Mediated Signaling in Myeloid Cells Is Required for Protective Innate Immunity During Pneumococcal Pneumonia
Source: Front Immunol. 2021 Sep 6;12:723967. doi: 10.3389/fimmu.2021.723967 (PMC8450579; doi:10.3389/fimmu.2021.723967)
Supplement: Supplementary file 1 [file DataSheet_1.pdf]

Figure S1

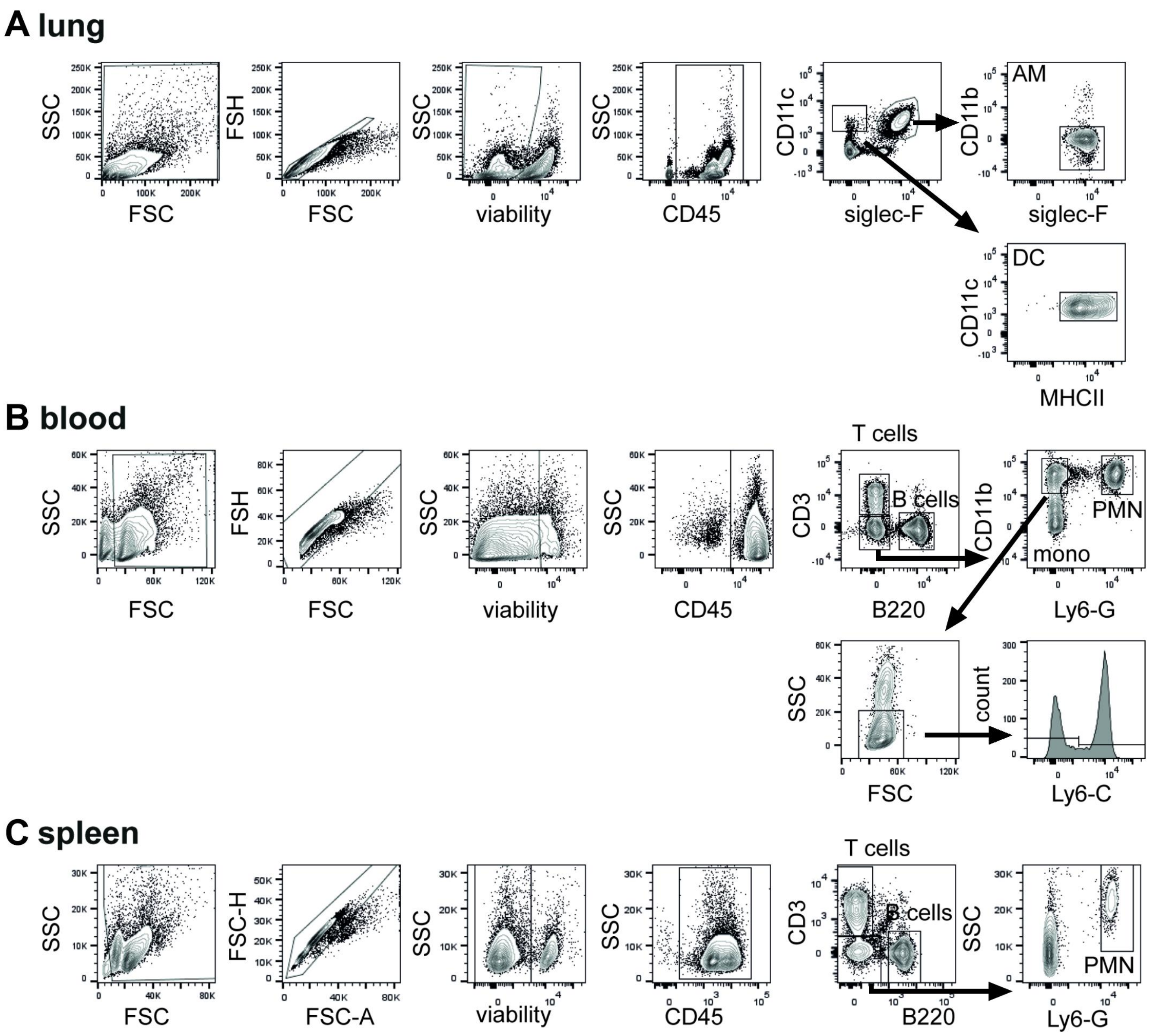

Figure S1: Gating strategy for myeloid cells and lymphocytes in lung, blood and spleen

Gating strategy for alveolar macrophages (Siglec-F^{+}/CD11c^{+}) and lung dendritic cells (Siglec-F^{-}/CD11c^{+}/MHCII^{+})(A), CD3^{+} T cells, B220^{+} B cells, Ly6G^{+}/CD11b^{+} neutrophils and Ly6G^{-}/CD11b^{+} monocytes (either Ly6C^{+} or Ly6C^{-}) in blood (B), and CD3^{+} T cells, B220^{+} B cells and Ly6G^{+}/CD11b^{+} neutrophils in spleen (C).

Figure S2

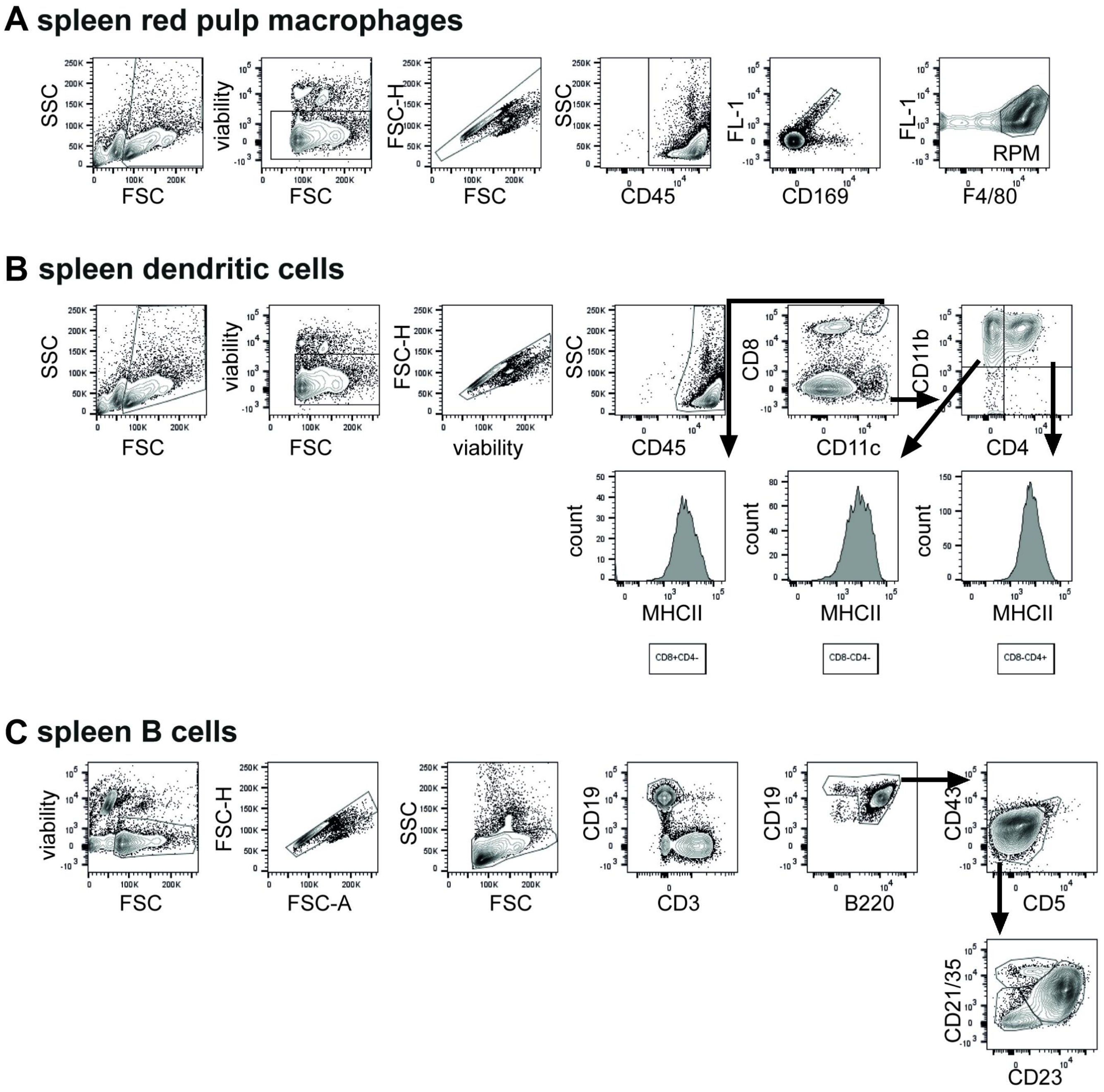

Figure S2: Gating strategy for myeloid cells and lymphocytes in spleen

Gating strategy for splenic red pulp macrophages (F4/80$^+$) (A), splenic MHCII$^+$CD11c$^+$CD8$^+$, MHCII$^+$CD11c$^+$CD4$^+$ and MHCII$^+$CD11c$^+$CD4$^-$CD8$^-$ dendritic cells (B), and splenic B cell subsets (Cd19$^+$/B220$^+$ cells (total B cells), CD5$^+$/CD43$^+$ B1 B cells, CD5^{lo}/CD43^{lo} B2 B cells, CD23$^+$ follicular B cells, CD21/35$^+$ marginal zone B cells and CD23^{lo}/CD21/35^{lo} transitional B cells (C).

Figure S3

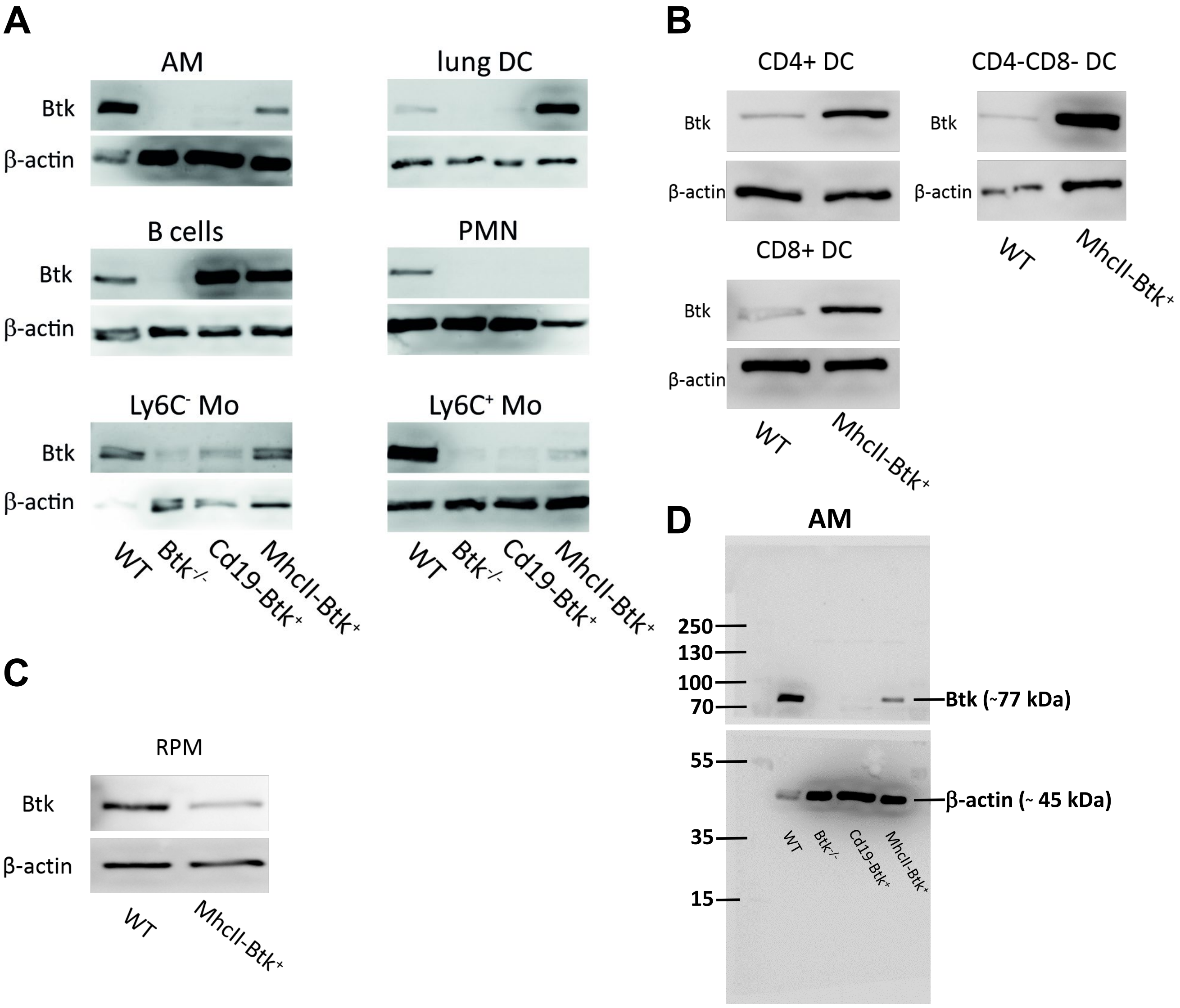

Figure S3 Analysis of Btk protein expression in sorted cells from lung, blood and spleen of WT and MhcII-Btk^{+} mice

Western blot analysis of Btk expression in sorted (A) alveolar macrophages (AM), lung dendritic cells (DC), and blood B cells, neutrophils (PMN), Ly6C^{-} and Ly6C^{+} monocytes, (B) splenic CD4+, CD8+ and CD4-CD8- dendritic cells and (C) splenic red pulp macrophages (RPM) of naïve WT, Btk$^{-/-}$, Cd19-Btk^{+} and MhcII-Btk^{+} mice. (D) Uncropped version of the Western blot of AM (from panel A) with molecular weight markers and deduced molecular weights of Btk and β-actin.

Figure S4

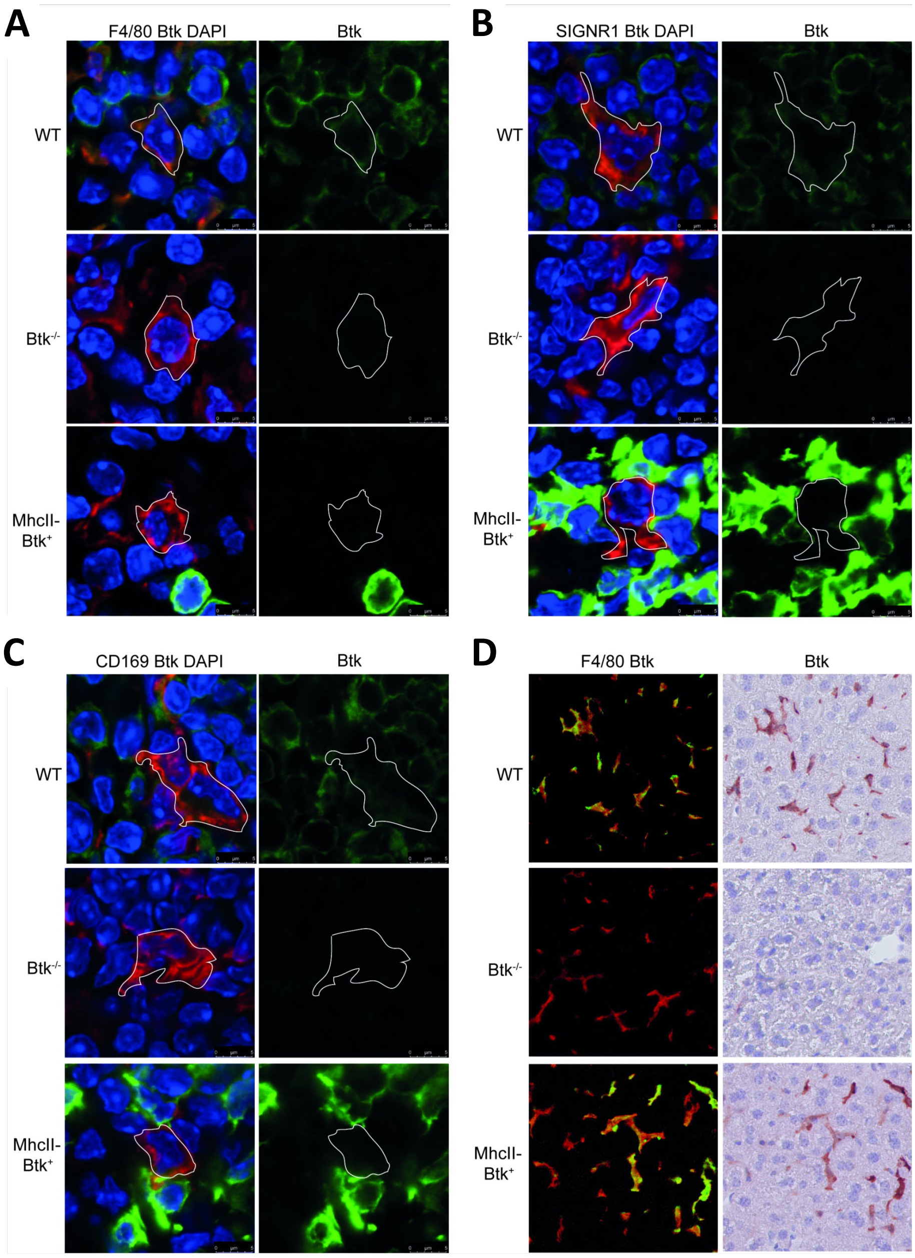

Figure S4: Btk expression of MhcII-Btk^{+} and WT mice in splenic and liver macrophages

Representative immunofluorescent staining of Btk in (A) F4/80^{+} red pulp macrophages, (B) SIGNR1^{+} marginal zone macrophages and (C) CD169^{+} marginal metallophilic macrophages in the spleen of naïve WT, Btk$^{-/-}$ and MhcII-Btk^{+} mice. Immunohistochemical staining of Btk in (D) F4/80^{+} Kupffer cells in liver of naïve WT, Btk$^{-/-}$ and MhcII-Btk^{+} mice. Btk staining is visible in WT spleen around macrophages and results most likely from Btk expression in B cells. This is supported by the finding that Btk is much stronger in the spleen of MhcII-Btk+ mice in the vicinity of marginal zone macrophages (CD169^{+} and SIGNR1^{+}), where B cells are located in the white pulp. Btk expression is very low in F4/80+ red pulp macrophages from WT mice and cannot be detected in MhcII-Btk^{+} mice and in CD169^{+} and SIGNR1^{+} cells of either WT and MhcII-Btk^{+} mice. The location of F4/80+ macrophages in the red pulp of the spleen and the location of CD169+ and SIGNR1+ macrophages in the marginal zone of the spleen is shown in Figure S6.

Figure S5

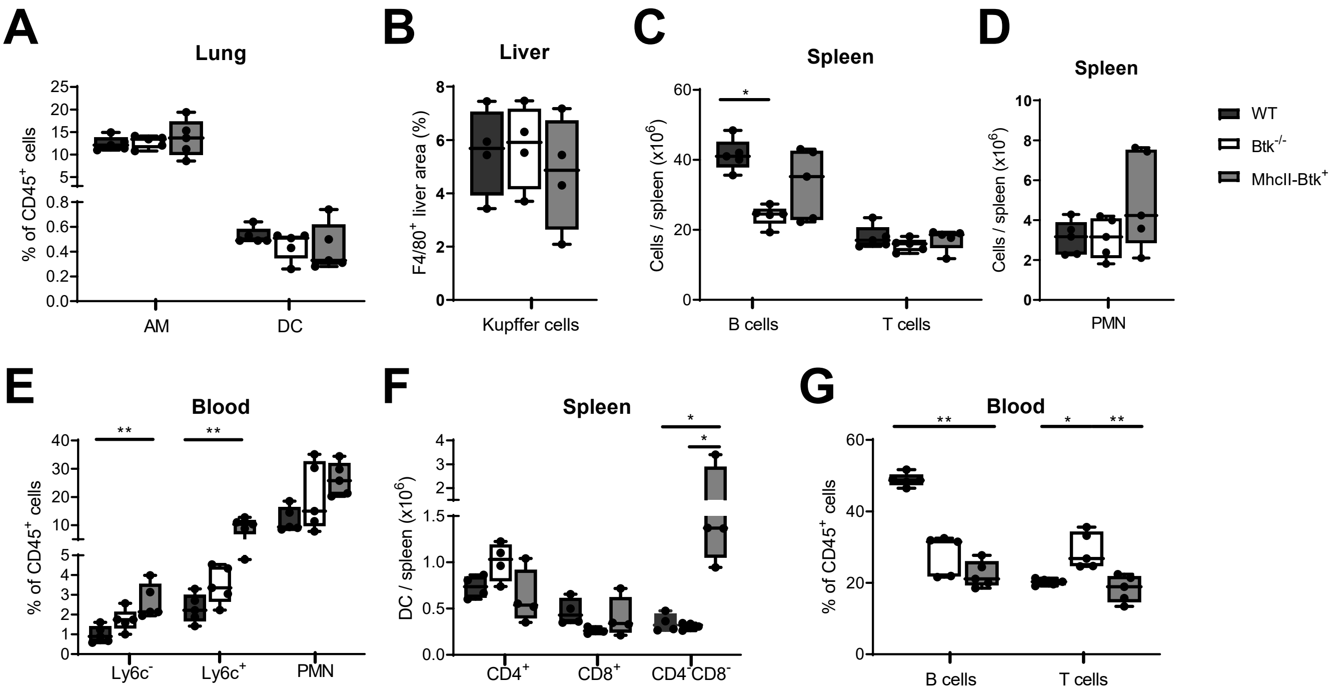

Figure S5: Analysis of cell composition in lung, blood, spleen and liver of WT and MhcII-Btk^{+} mice

Naïve WT, Btk$^{-/-}$ and MhcII-Btk^{+} mice (n=5 per group) were sacrificed. (A) Percentage of alveolar macrophages (AM) and dendritic cells (DC) in the lung and (B) Kupffer cells in the liver. (C) Total B and T lymphocyte and (D) neutrophil (PMN) numbers in the spleen. (E) Percentage of Ly6C^{-} and Ly6C^{+} monocytes and neutrophils in blood. (F) total numbers of DC subsets in the spleen. (G) Percentage of B and T lymphocytes in the blood. Data are expressed as box- and whisker plots showing the smallest observation, lower quartile, median, upper quartile and largest observation, and individual data points. *p<0.05, **p<0.01, Kruskall-Wallis test with false discovery rate correction.

Figure S6

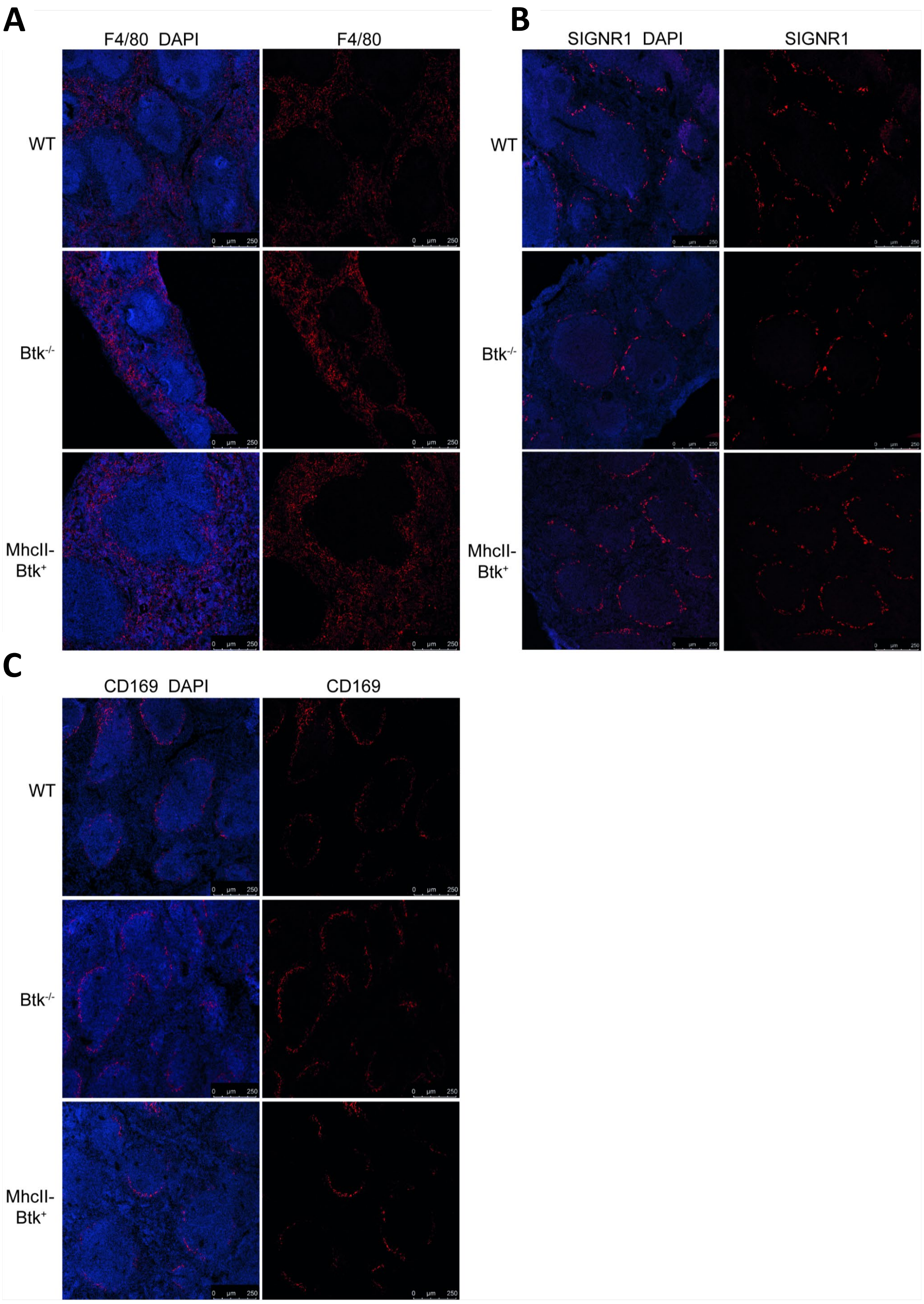

Figure S6: Overview of red pulp, marginal zone and metallophilic macrophages in the spleen of WT and MhcII-Btk^{+} mice

Representative overview of (A) F4/80^{+} red pulp macrophages (B) SIGNR1^{+} marginal zone macrophages (C) and CD169^{+} marginal metallophilic macrophages in the spleen of naïve mice.

Figure S7

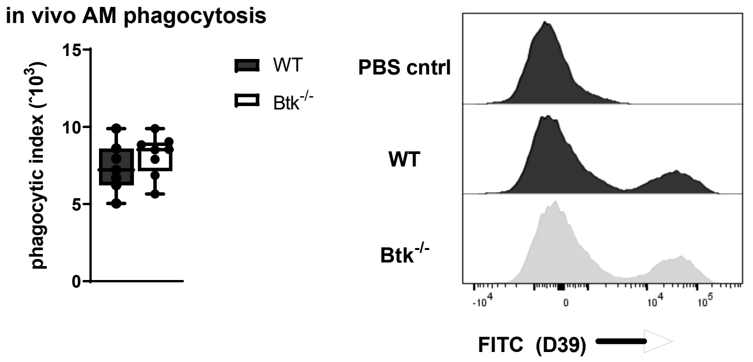

Figure S7: In vivo phagocytosis of alveolar macrophages from WT and Btk$^{-/-}$ mice

The phagocytic index of alveolar macrophages of WT (n=7) and Btk$^{-/-}$ (n=8) mice 4 hours after intranasal inoculation with heat killed FITC labelled D39. Data are expressed as box- and whisker plots showing the smallest observation, lower quartile, median, upper quartile and largest observation, and individual data points. Representative histograms are shown of D39 uptake by WT and Btk$^{-/-}$ alveolar macrophages.

Figure S8

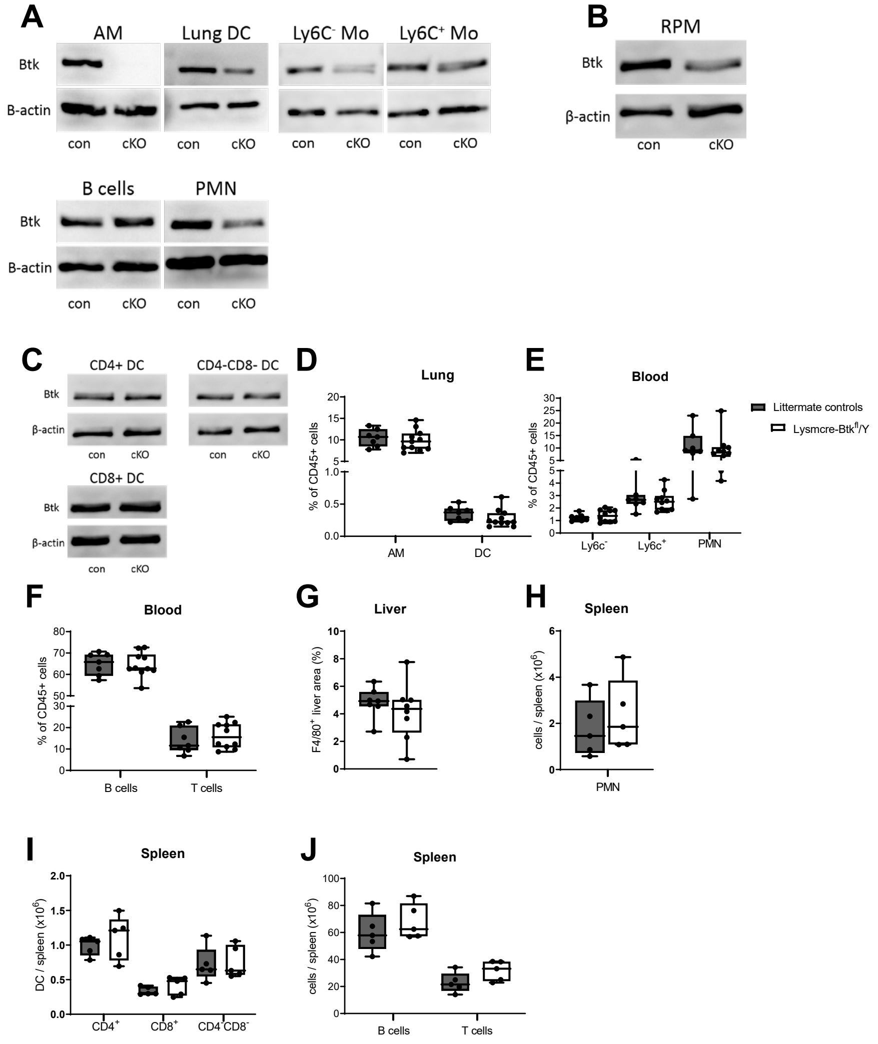

Figure S8: Analysis of Btk protein expression in sorted macrophages, neutrophils, monocytes and dendritic cells and cell composition in lung, blood, spleen and liver in Lysmcre-Btk^{fl}/Y and littermate controls

(A) Western blot analysis of Btk expression in sorted lung myeloid cells and blood leukocytes and in splenic (B) red pulp macrophages and (C) dendritic cells of naïve Lysmcre-Btk^{fl}/Y mice (cKO) and littermate controls (con). For cell composition naïve Lysmcre-Btk^{fl}/Y and littermate controls (n=7-10 per group) were sacrificed. (D) Percentage of alveolar macrophages (AM) and dendritic cells (DC) in the lung and (E) Ly6C^{-} and Ly6C^{+} monocytes and neutrophils (PMN) in blood. (F) Percentage of B and T lymphocytes in blood. (G) Percentage of Kupffer cells. (H) Total number of neutrophils (PMN) in the spleen. (I) Total numbers of DC subsets in the spleen. (J) total B cell and T cell numbers in the spleen. Data are expressed as box- and whisker plots showing the smallest observation, lower quartile, median, upper quartile and largest observation, and individual data points.

Figure S9

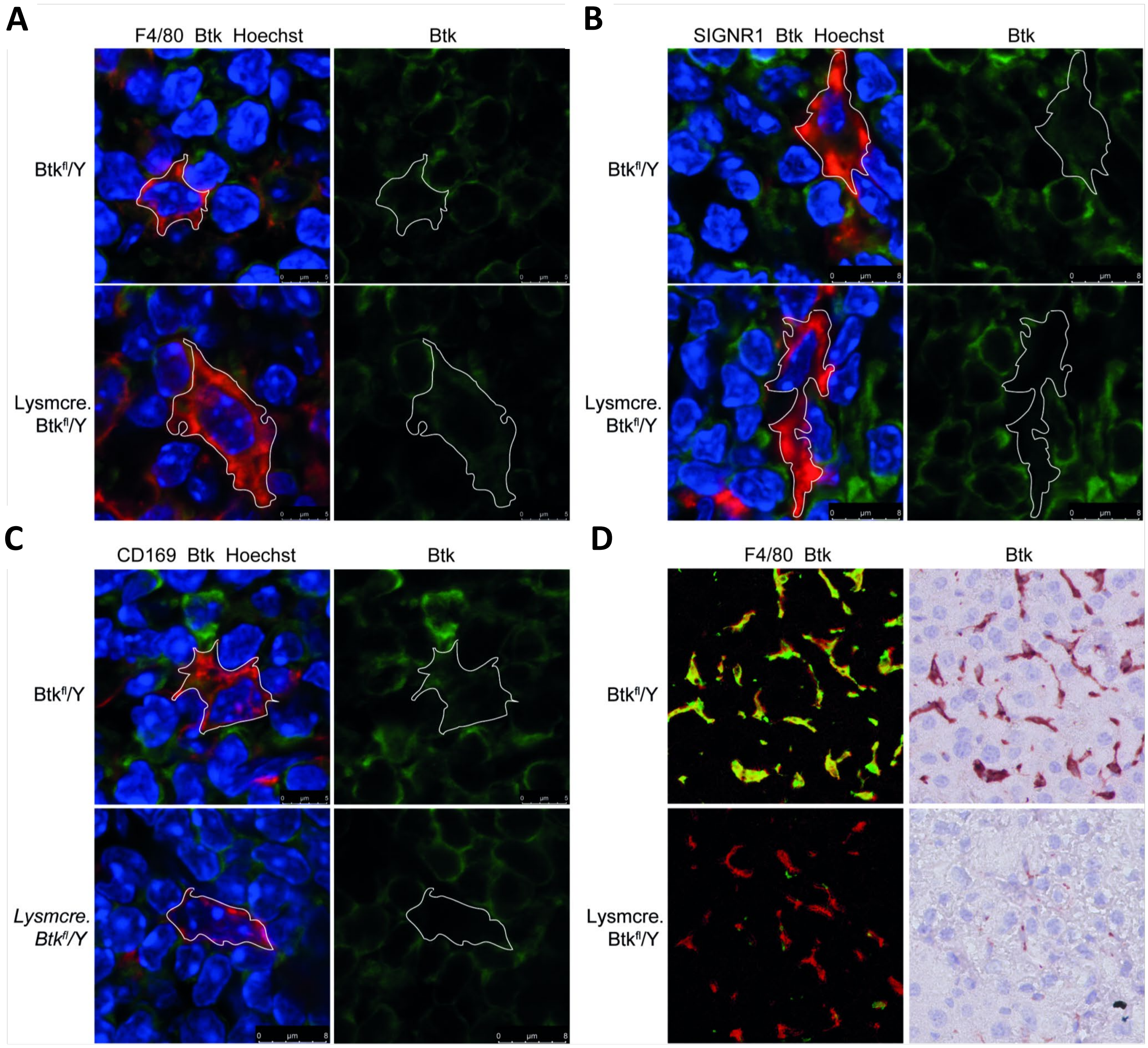

Figure S9: Btk expression of Lysmcre-Btk^{fl}/Y and littermate control mice in splenic and liver marcophages

Immunofluorescent staining of Btk in (A) F4/80^{+} red pulp macrophages, (B) SIGNR1^{+} marginal zone macrophages and (C) CD169^{+} marginal metallophilic macrophages in the spleen of naïve Lysmcre-Btk^{fl}/Y and littermate control mice. Immunohistochemical staining of Btk in (D) F4/80^{+} Kupffer cells of naïve Lysmcre-Btk^{fl}/Y and littermate control mice. Btk staining is visible in spleen of littermate controls around CD169^{+} and SIGNR1^{+} marginal zone macrophages and results most likely from Btk expression in B cells. Btk expression is very low in all macrophage types in spleen of control mice and cannot be detected in Lysmcre-Btk^{fl}/Y mice. The location of F4/80+ macrophages in the red pulp of the spleen and the location of CD169+ and SIGNR1+ macrophages in the marginal zone of the spleen is shown in Figure S10.

Figure S10

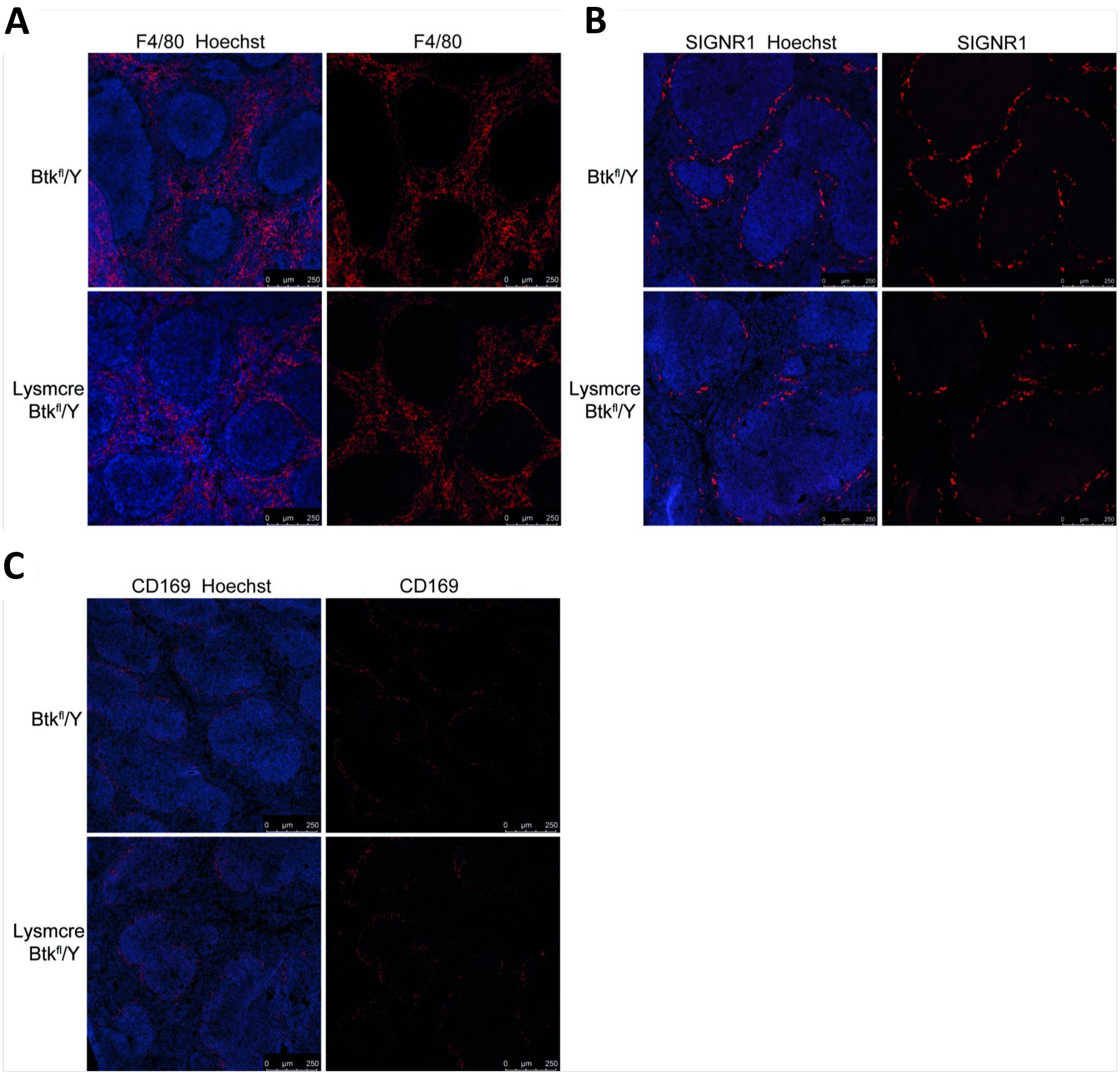

Figure S10: Overview of red pulp, marginal zone and marginal metallophilic macrophages in the spleen of littermate control and Lysmcre-Btk^{fl}/Y mice

Representative overview of (A) F4/80^{+} red pulp macrophages (B) SIGNR1^{+} marginal zone macrophages (C) and CD169^{+} marginal metallophilic macrophages in the spleen of naïve mice.

Figure S11

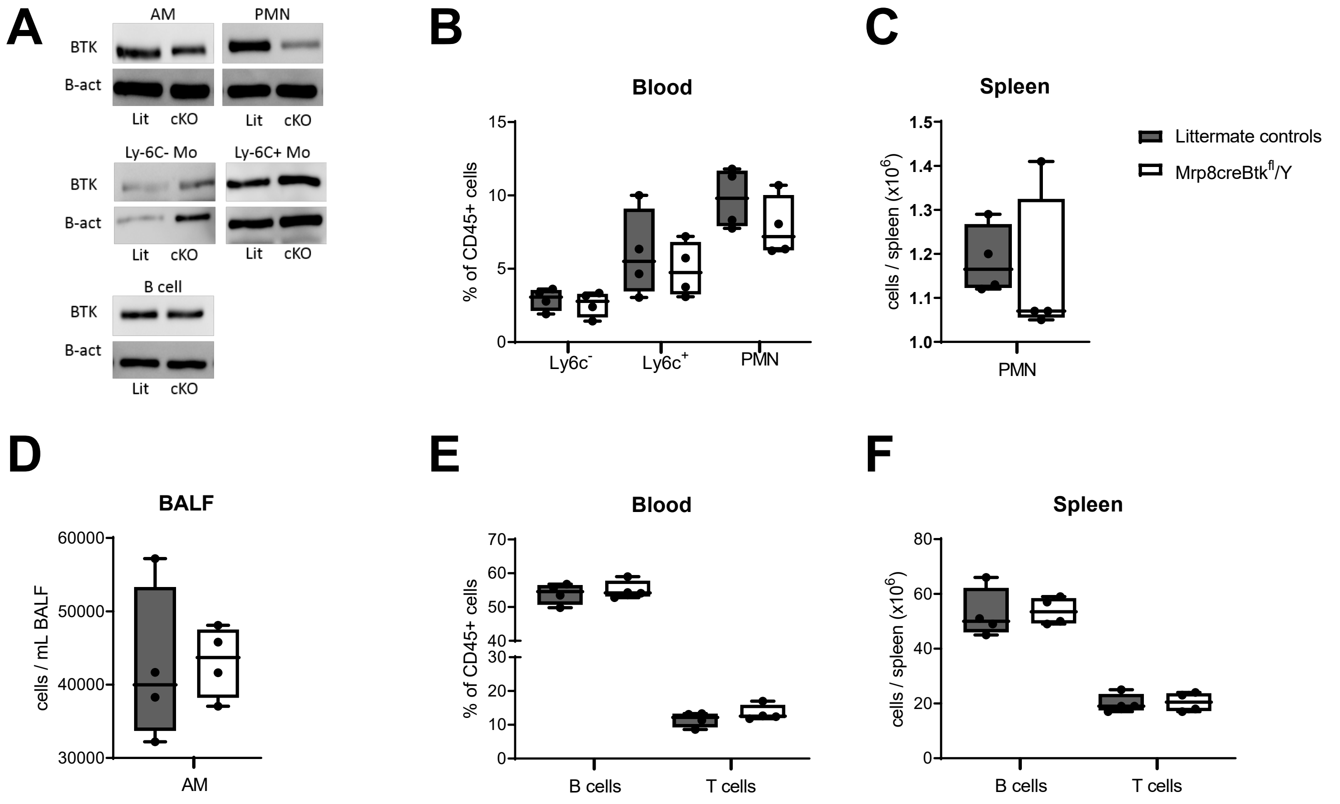

Figure S11: Analysis of Btk protein expression in sorted macrophages, neutrophils, monocytes and dendritic cells and cell composition in lung, blood, spleen and liver in Mrp8cre-Btk^{fl}/Y and littermate controls

(A) Western blot analysis of Btk expression in sorted lung myeloid cells and blood leukocytes and of naïve Mrp8cre-Btk^{fl}/Y mice (cKO) and littermate controls (con). For cell composition naïve Mrp8cre-Btk^{fl}/Y and littermate controls (n=4 per group) were sacrificed. (B) Ly6C^{-} and Ly6C^{+} monocytes and neutrophils (PMN) in blood and (C) spleen. (D) Percentage of alveolar macrophages (AM) in bronchoalveolar lavage fluid (BALF). (E) Percentage of B and T lymphocytes in blood and (F) total B cell and T cell numbers in the spleen. Data are expressed as box- and whisker plots showing the smallest observation, lower quartile, median, upper quartile and largest observation, and individual data points.

Figure S12

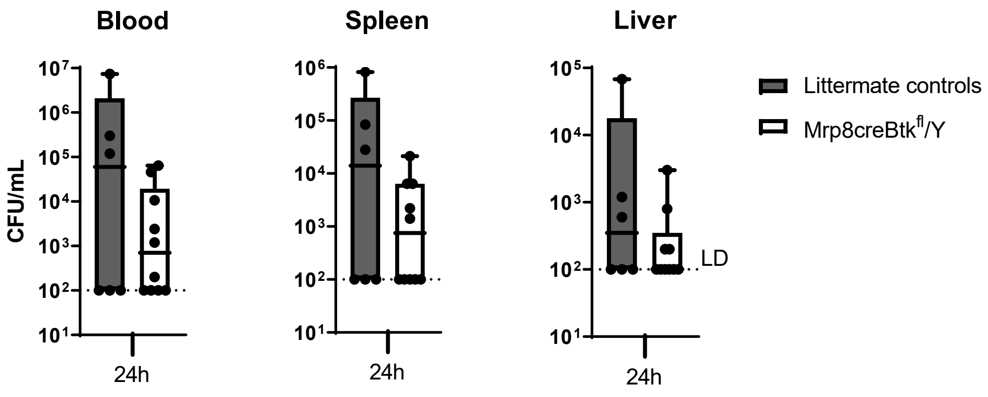

Figure S12: Bacterial loads in blood, spleen and liver of Mrp8-creBtk^{fl}/Y (n=10) and littermate contols (n=6) 24 hours after intravenous injection with D39.

Data are expressed as box- and whisker plots showing the smallest observation, lower quartile, median, upper quartile and largest observation, and individual data points.

Figure S13

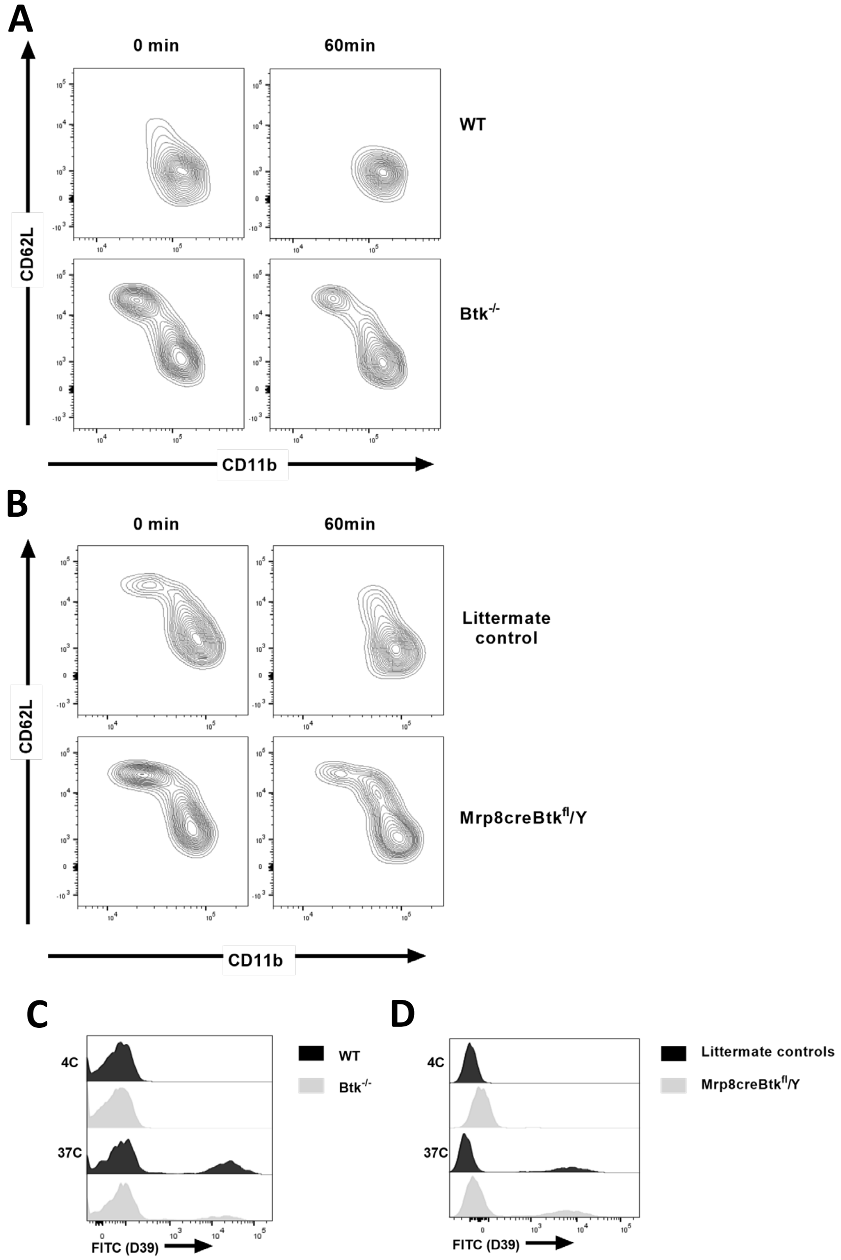

Figure S13: Contour plots of neutrophil CD11b and CD62L expression and histograms of neutrophil phagocytosis after whole blood stimulation with streptococcus D39

(A,B) Representative contour plots of CD11b and CD62L expression on WT and Btk$^{-/-}$ and littermate control and Mrp8cre-Btk^{fl}/Y neutrophil are shown after whole blood stimulation for 0 and 60 minutes with heat killed D39. (C,D) Representative histograms of FITC positivity of littermate control, Mrp8creBtk^{fl}/Y, WT and Btk$^{-/-}$ neutrophils after 60 minutes of whole blood incubation at 4°C and 37°C with heat killed FITC labelled D39.

Table S1 Comparison of Btk expression patterns in humans and in mouse strains used in this study

	B cells	Monocytes	Alveolar Macrophages	Granulocytes
Human*	+	+	+	+
WT mice**	+	+	+	+
$Btk^{-/-}$ mice**	-	-	-	-
CD19-Btk^{+} ($Btk^{-/-}$) mice**	+	-	-	-
MhcII-Btk^{+} ($Btk^{-/-}$) mice**	+	+/-	+/-	-
Lysmcre-Btk^{fl}/Y mice**	+	+/-	-	+/-
Mrp8cre-Btk^{fl}/Y mice**	+	+	+	+/-

* based on BTK mRNA expression data from the Human Protein Atlas
** based on Western blot analysis of samples obtained from C57BL6 mice in this study
+ present; - absent; +/- reduced (as compared to WT)

Table S2 Antibodies used for flow cytometry

Antibody	Conjungate	Clone	Company	Cat. No
CD45R/B220	FITC	RA3-6B2	BD Bioscience	553087
CD4	FITC	RM4-5	BD Biosciences	553047
CD3	PerCP-CY5.5	17A2	BD Biosciences	560527
CD11c	PerCP-CY5.5	HL3	BD Biosciences	560584
CD8a	APC	53-6.7	BD Biosciences	553035
Siglec F	Alexa647	E50-2440	BD Bioscience	562680
CD11b	PE Cy7	M1/70	BD Bioscience	561098
LY6G	PE	1A8	BD bioscience	551461
LY6G	APC	1A8	BD Bioscience	560566
LY6G	Alexa fluor 700	1A8	BD Bioscience	561236
LY6C	Alexa fluor 700	AL-21	BD bioscience	561237
CD11c	FITC	HL3	BD Bioscience	553801
CD11c	BV421	HL3	BD Biosciences	562782
CD62L	APC	MEL-14	BD Biosciences	553152
MHCII	Alexa fluor 700	M5/114.15.2	ThermoFisher	56-5321-80
CD5	PerCP-CY5.5	53-7.3	ThermoFisher	45-0051-80
CD45	PE efluor610	30-F11	ThermoFisher	61-0451
CD23	PE Cy7	B3B4	ThermoFisher	25-0232-81
CD169	efluor660	SER-4	ThermoFisher	50-5755-80
CD21/135	APC	7G6	ThermoFisher	561770
F4/80	Alexa fluor 700	BM8	ThermoFisher	56-4801-82
CD43	PE	ebioR2/60	ThermoFisher	12-0431-81
CD19	PE-efluor610	eBio1D3	ThermoFisher	61-0193-80
CD3	Alexa fluor 700	17A2	ThermoFisher	56-0032-80
Fixable viability dye	eFluor 780	-	ThermoFisher	65-0865-14
CD16/32	-	93	ThermoFisher	14-0161-82

Table S3 Plasma IgM and IgG3 natural antibodies in naive WT, Btk$^{-/-}$ and Cd19-Btk^{+} and MhcII-Btk^{+} mice.

**IgM**	**WT**	**Btk$^{-/-}$**	**Cd19-Btk^{+}**	**MhcII-Btk^{+}**
Phosphocholine	0.23 (0.11-0.26)	0.08 (0.03-0.12)	0.28 (0.20-0.50)†	0.52 (0.26-0.61)†††
S.pneumoniae D39	0.39 (0.35-0.47)	0.14 (0.06-0.19)	0.76 (0.63-0.86)††	0.75 (0.55-0.94)†††
Capsule ST2	0.54 (0.46-0.79)	0.01 (0-0.02)*	0.83 (0.66-0.89)††	0.60 (0.50-0.89)††
S.pneumoniae WU2	0.28 (0.22-0.29)	0.05 (0.04-0.07)	0.64 (0.44-0.95)*; †††	0.62 (0.52-0.79)*; †††
Capsule ST3	0.14 (0.10-0.18)	0.06 (0.05-0.10)	0.45 (0.32-0.75)*; †††	0.50 (0.35-0.81)*; †††
**IgG3**	**WT**	**Btk$^{-/-}$**	**Cd19-Btk^{+}**	**MhcII-Btk^{+}**
Phosphocholine	0.17 (0.11-0.21)	0.05 (0.04-0.07)*	0.15 (0.10-0.28) †	0.29 (0.27-0.40) ††††
S.pneumoniae D39	0.50 (0.43-0.59)	0 (0-0.04)*	0.56 (0.41-0.61) †	0.60 (0.54-0.66) †††
Capsule ST2	0.12 (0.11-0.24)	0.01 (0-0.03)*	0.31 (0.07-0.38) †††	0.23 (0.13-0.48) †††
S.pneumoniae WU2	0.37 (0.33-0.48)	0.29 (0.26-0.32)	0.54 (0.49-0.61) ††	0.66 (0.52-0.71)**; †††
Capsule ST3	0.14 (0.13-0.21)	0.07 (0.06-0.11)*	0.18 (0.13-0.22) †	0.23 (0.19-0.39)*; †††

*= compared to WT, †=compared to *Btk$^{-/-}$*.
*/† p<0.05; **/†† p<0.01; ††† p<0.001; †††† p<0.0001, Kruskall-Wallis test with false discovery rate correction.
Data are presented as median (interquartile range) optical density (OD) of 450nm-650nm of groups of n=7-8 mice.

Table S4 Plasma IgM and IgG3 natural antibodies in WT, Btk$^{-/-}$ and Cd19-Btk^{+} 24 hours after intravenous infection with D39 or WU2.

***S.pneumoniae* D39**	**WT**	**Btk$^{-/-}$**	**Cd19-Btk^{+}**
IgM	0.75 (0.74-0.80)	0.19 (0.14-0.22)***	0.72 (0.69-0.75) †
IgG3	0.44 (0.40-0.47)	0.28 (0.25-0.29)***	0.35 (0.33-0.43) †
***S.pneumoniae* WU2**	**WT**	**Btk$^{-/-}$**	**Cd19-Btk^{+}**
IgM	0.79 (0.74-0.82)	0.04 (0.03-0.05)*	0.91 (0.90-0.91)*; ††††
IgG3	0.54 (0.53-0.55)	0.32 (0.31-0.35)**	0.55 (0.48-0.59) ††

*= compared to WT, †=compared to *Btk$^{-/-}$*.
*/† p<0.05; **/†† p<0.01; *** p<0.001; †††† p<0.0001, Kruskall-Wallis test with false discovery rate correction.
Data are presented as median (interquartile range) optical density (OD) of 450nm-650nm of groups of n=6-8 mice.

Table S5 Lung cytokines in WT, Btk$^{-/-}$ and MhcII-Btk^{+} mice 48 hours after intranasal infection with D39.

	**48h**		
	**WT**	***Btk$^{-/-}$***	***MhcII-Btk^{+}***
TNF (pg/mL)	125 (125-125)	902 (369-1415)****	132 (125-275) ††
IL-6 (pg/mL)	<LD	1745 (581-2353)***	63 (63-337) ††
IL-1β (pg/mL)	<LD	174 (92-229)**	63 (63-80)

*= compared to WT, †=compared to *Btk$^{-/-}$*
**/†† p<0.01; *** p<0.001; **** p<0.0001, Kruskall-Wallis test with false discovery rate correction.
LD = limit of detection; 63 pg/ml. Data are presented as median (interquartile range) of groups of n=8 mice.

Table S6 Cytokines and chemokines in supernatant of alveolar macrophages 24 hours after stimulation with D39.

	**WT**	**Btk$^{-/-}$**
IL-6 (pg/mL)	15 (11-18)	3 (3-5)***
CXCL2 (pg/mL)	2419 (2085-2903)	1891 (1675-2058)*
CXCL1 (pg/mL)	2825 (2186-4294)	1756 (1530-1989)**

* p<0.05; ** p<0.01; *** p<0.001, Mann-Whitney U test.
Data are presented as median (interquartile range) of groups of n=8 mice.

Table S7 Plasma IgM and IgG3 D39 specific natural antibodies in naive LysMcreBtk^{fl}/Y mice and littermate controls.

	Littermate control	LysMcreBtk^{fl}/Y
IgM	0.46 (0.29-0.58)	0.35 (0.26-0.62)
IgG3	0.51 (0.44-0.64)	0.53 (0.46-0.58)

Data are presented as median (interquartile range) optical density (OD) of 450nm-650nm of groups of n=8 mice.

Table S8 BALF cytokines and chemokines in LysMcreBtkfl/Y mice and littermate controls 3 hours after antranasal infection with D39.

	Littermate control	LysMcreBtk^{fl}/Y
IL-6 (pg/mL)	<LD	63 (63-68)
CXCL2 (pg/mL)	1402 (1143-1656)	1205 (947-1567)
CXCL1 (pg/mL)	1176 (1086-1816)	1404 (1087-1527)

Limit of detection; 63pg/mL. Data are presented as median (interquartile range) of groups of n=8 mice.

Table S9 Lung cytokines in LysMcreBtk^{fl}/Y mice and littermate controls 48 hours after intranasal infection with D39.

	Littermate control	LysMcreBtk^{fl}/Y
TNF (pg/mL)	5746 (3916-11124)	3559 (1861-3815)*
IL-6 (pg/mL)	2770 (2207-4135)	1670 (1391-2504)
IL-1β (pg/mL)	2539 (2288-3623)	2715 (1978-4439)

* p<0.05, Mann-Whitney U test.
Data are presented as median (interquartile range) of groups of n=7-8 mice.

Table S10 Plasma IgM and IgG3 D39-specific natural antibodies in naive Mrp8creBtk^{fl}/Y mice and littermate controls.

	Littermate control	Mrp8creBtk^{fl}/Y
IgM	0.68 (0.55-0.83)	0.49 (0.44-0.70)
IgG3	0.57 (0.53-0.62)	0.55 (0.48-0.60)

Data are presented as median (interquartile range) optical density (OD) of 450nm-650nm of groups of n=5-7 mice.

Table S11 Lung cytokines in Mrp8creBtk^{fl}/Y mice and littermate controls 41 hours after intranasal inoculation with D39.

	Littermate control	Mrp8creBtk^{fl}/Y
TNF (pg/mL)	646 (311-1232)	1178 (584-2017)
IL-6 (pg/mL)	731 (416-1579)	2668 (1330-7701)*
IL-1β (pg/mL)	896 (630-1215)	1387 (773-2076)

* p<0.05, Mann-Whitney U test.
Data are presented median (interquartile range) of groups of n=10-14 mice.

Table S12 Plasma IgM and IgG3 natural antibodies in naive littermate controls and Cd19creBtkfl/Y mice and in Btk$^{-/-}$ mice.

IgM	Littermate control	Cd19creBtk^{fl}/Y	Btk$^{-/-}$
S.pneumoniae D39	0.93 (0.58-0.97)	0.91 (0.57-0.95)	0.14 (0.10-0.25) *; †
S.pneumoniae WU2	0.98 (0.78-1.05)	0.96 (0.90-0.98)	0.21 (0.15-0.29) *; †
Capsule ST2	0.95 (0.91-0.99)	0.93 (0.92-0.96)	0.09 (0.08-0.17) *; †
Capsule ST3	0.67 (0.56-0.77)	0.53 (0.32-0.59)	0.10 (0.08-0.18) **
Phosphocholine	1.02 (0.94-1.05)	1.01 (0.90-1.03)	0.15 (0.12-0.31) *
**IgG3**	**Littermate control**	**Cd19creBtk^{fl}/Y**	**Btk$^{-/-}$**
S.pneumoniae D39	0.46 (0.31-0.49)	0.40 (0.37-0.44)	0.14 (0.13-0.26) *; †
S.pneumoniae WU2	0.34 (0.32-0.50)	0.31 (0.28-0.37)	0.23 (0.21-0.25) **; †
Capsule ST2	0.19 (0.17-0.23)	0.18 (0.15-0.21)	0.09 (0.07-0.11) *; †
Capsule ST3	0.18 (0.17-0.20)	0.14 (0.13-0.17)	0.10 (0.10-0.14) **
Phosphocholine	0.20 (0.17-0.28)	0.22 (0.16-0.26)	0.11 (0.08-0.15) *; †

*= compared to Littermate WT controls, †=compared to Cd19cre-Btk^{fl}/Y.

*/† p<0.05; ** p<0.01, Kruskall-Wallis test with false discovery rate correction.

Data are presented as median (interquartile range) optical density (OD) of 450nm-650nm of groups of n=5 mice.
